# Comprehensive causal analysis between autoimmune diseases and glioma: A Mendelian randomization study

**DOI:** 10.1097/MD.0000000000041815

**Published:** 2025-03-07

**Authors:** Ni Deng, Rafeq Agila, Qiang He, Chao You, Songping Zheng

**Affiliations:** a Department of Respiratory Therapy, West China Hospital, Sichuan University, Chengdu, China; b Department of Neurosurgery, West China Hospital, Sichuan University, Chengdu, Sichuan, China.

**Keywords:** autoimmune diseases, causal association, Glioma, subtypes

## Abstract

The causal association between the autoimmune disease and the development of glioma and its subtypes remains unclear. We performed a comprehensive Mendelian randomization (MR) to clarify their causal association from genetic perspective. We obtained the summary-level datasets for autoimmune diseases from recently published genome-wide association studies in the UK Biobank (UKB) and the FinnGen consortium. Additionally, we collected summary statistics datasets related to glioma and its subtypes from a comprehensive meta-analysis genome-wide association study, which included 12,488 cases and 18,169 controls. We primarily used inverse variance weighting method, supplemented by Bonferroni correction to account for multiple tests to reduce the probability of false positive results. We also performed sensitivity analyses to address potential pleiotropy and strengthen the reliability of the results. After meta-analysis, pernicious anemia may decrease the risk of glioblastoma (GBM) (UKB: odds ratio (OR) = 0.01, 95% confidence interval (CI) = 0.01–0.02, *P* = 1.01E‐12; FinnGen: OR = 0.86, 95% CI = 0.79–0.93, *P* = .0002; Meta: OR = 0.04, 95% CI = 0.03–0.04). In reverse MR analysis, GBM decreased the risk of celiac disease (UKB: OR = 0.96, 95% CI = 0.95–0.98, *P* = .0000; FinnGen: OR = 0.89, 95% CI = 0.84–0.94, *P* = .0001; Meta: OR = 0.95, 95% CI = 0.94–0.97). Heterogeneity and pleiotropy analyses, and reverse analysis, confirmed the robustness of these results. From the genetic perspective, our MR study uncovered that pernicious anemia may decrease the risk of GBM. Conversely, GBM appeared to mitigate the risk of celiac disease. Future studies are required to validate the causal association and illuminate the underlying mechanisms.

## 1. Introduction

It is widely accepted that the immune system plays a crucial role in protecting the brain from infections. Autoimmune manifestations are observed in various diseases, including encephalitis, multiple sclerosis, Alzheimer disease, and certain rare tumors.^[1,2]^ One possible explanation is that the hypersensitive immune system, which is active against transformed cells, predisposes individuals to asthma. However, these studies are often affected by self-report bias, as many fail to verify asthma diagnoses among both cases and controls. Many studies have explored the relationship between the risk of glioma and immune-related conditions. The observed protective role of allergies on glioma contrasts with the general association between autoimmune conditions and cancer risk.^[3,4]^ Atopic disease also had a strong inverse relationship with glioma in a meta-analysis of 3450 patients diagnosed with glioma.^[5]^ In contrast, some observational studies, including 1 involving 880 subsequent brain cancers, reported no significant relationship between autoimmune diseases and glioma risk.^[6]^ A study including a total of 138,723 from the Swedish Cancer Registry found no association between hay fever or allergic rhinitis and glioma development.^[7]^ Notably, we should interpret these findings with caution due to the limitations inherent in observational research, such as potential confounding factors, retrospective study designs, and limited sample sizes, all of which may impact the reliability of the conclusions drawn. In the field of therapy, despite the proven efficacy of immunotherapy in certain solid tumors, such as lung cancer and melanoma,^[8,9]^ its application in glioblastoma has yet to yield significant improvements in patient survival.^[10]^ Therefore, a deeper understanding of the mechanisms of immune modulation in glioma and its subtypes is essential for the development of novel and more effective immunotherapeutic strategies. Given that glioma is a relatively rare disease,^[11]^ doubts have been raised regarding the robustness of conclusions drawn from these studies.

To address the inconsistent results in observational design, Mendelian randomization (MR) is a robust method employing genetic variants (single-nucleotide polymorphisms, SNPs) to investigate causal relationships between exposures and glioma.^[12]^ SNPs, governed by meiotic randomness during zygote formation in gestation,^[13,14]^ make MR analysis unaffected by the impact of diseases. MR acts akin to a randomized controlled trial, efficiently grouping subjects without the extensive resources required for such trials, and can avoid reverse causation and overcome confounding factors that are typical of non-randomized observational studies.^[15]^ Unlike observational studies, MR mitigates confounder influences common in traditional observational studies, and overcomes the endogeneity and then yield causal estimates in this study. MR analysis usually use SNPs from genome-wide association studies (GWAS) associated with diseases as genetic instruments to examine the causal association between the exposures (i.e., autoimmune diseases) and outcomes (i.e., glioma). Despite this, no MR studies have investigated the association between autoimmune disease and glioma and its subtypes. We initially performed comprehensive MR, reverse MR, and replicate MR to explore the causal relationship between 25 autoimmune diseases and glioma and its subtypes. Therefore, this two-sample MR study aims to uncover potential association, thereby enhancing our understanding of the intricate involvement of the autoimmune diseases in glioma risk.

## 2. Methods

### 2.1. Ethical approval and study design

**Figure 1.**
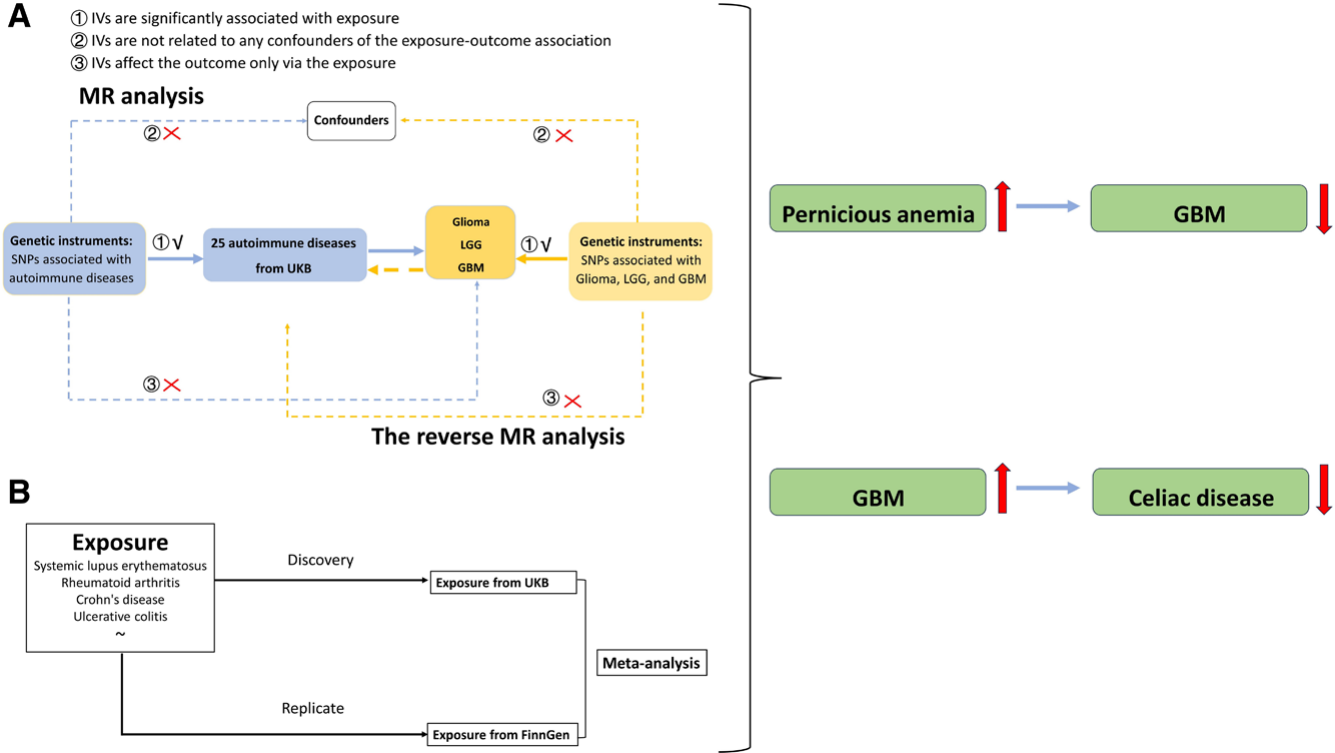
Study design for identifying autoimmune diseases causally associated with glioma, LGG, and GBM. LGG = low-grade glioma, GBM = glioblastoma.

We obtained the summary-level statistics for both exposure and outcome datasets from de-identified datasets of previously approved studies, sourced from GWAS databases focusing on European ancestry. Ethical approval and participant informed consent were obtained from the relevant ethics committees. As this MR study solely utilizes summary-level datasets, it is exempt from further ethical approval. Furthermore, this study conforms to the Strengthening the Reporting of Observational Studies in Epidemiology using Mendelian Randomization checklist for comprehensive reporting standards in observational studies,^[16,17]^ and the study flowchart is displayed in Figure 1. All the genetic variants to qualify as valid instrumental variables must meet 3 assumptions: (1) instrumental variables (IVs) are significantly associated with exposure; (2) IVs are not related to any confounders of the exposure-outcome association; and (3) IVs affect the outcome only via the exposure.^[18]^

### 2.2. Data sources of autoimmune diseases

We collected a total of 25 autoimmune diseases from UK Biobank (UKB), including systemic lupus erythematosus, rheumatoid arthritis, Crohn disease, ulcerative colitis, type 1 diabetes, celiac disease, psoriasis, ankylosing spondylitis, multiple sclerosis, primary biliary cholangitis, asthma, eczema or dermatitis, amyotrophic lateral sclerosis, Bechet disease, graves’ disease, Hashimoto thyroiditis, idiopathic thrombocytopenic purpura, myasthenia gravis, pernicious anemia, polymyalgia rheumatic, rheumatic fever, sarcoidosis, systemic sclerosis, Wegener granulomatosis, and adrenocortical insufficiency. All participants had a European genetic background in the MR study. In addition, we also performed replicated MR analysis using 25 exposures from the FinnGen consortium. Detailed information on the exposures is displayed in Table S1, Supplemental Digital Content, http://links.lww.com/MD/O499. All summary-level datasets from UKB have been published previously, which had a comprehensive understanding of these exposure adjustments.^[19–36]^ Autoimmune diseases from the FinnGen consortium have been defined using the codes of the International Classification of Diseases (ICD-9) and ICD-10. Age, sex, and genetic principal components have been adjusted.^[37]^ In addition, undefined sex, high genotype deletion (>5%), excess heterozygosity (±4 standard deviations), and non-Finnish ancestry were not included.

### 2.3. Data sources of glioma, LGG, and GBM

We collected the summary-level datasets for glioma, including lower-grade glioma (LGG, non-GBM), and glioblastoma (GBM) from a recent meta-analysis involving 8 glioma GWAS.^[38]^ This comprehensive meta-analysis encompassed a substantial cohort of 12,488 cases and 18,169 controls (Table S1, Supplemental Digital Content, http://links.lww.com/MD/O499 and Table S2, Supplemental Digital Content, http://links.lww.com/MD/O500). The classification of gliomas was based on malignancy grade, distinguishing between pilocytic astrocytoma (World Health Organization [WHO] grade I), diffuse “low-grade” glioma (WHO grade II), anaplastic glioma (WHO grade III), and GBM (WHO grade IV). Further categorization included LGG (non-GBM, N = 5820) and GBM (N = 6183). It is noteworthy that all datasets originated from European ancestry, and there was no overlap between the exposure and outcome datasets. Glioma, LGG, and GBM were obtained from GLIOGENE Consortium.

### 2.4. Instrumental variables selection

Initially, we conducted a rigorous filtering process to collect candidate single SNPs with a strong association with exposures as IVs. This selection was at a genome-wide significance level, meeting a *P*-value of <5 × 10^‐8^, indicting a robust association with the exposure. To mitigate the impact of linkage disequilibrium, we employed an *r*^2^ < 0.001 threshold and a clumping window of 10,000 kb to ensure their independence based on the European population as a reference. These criteria enabled us to obtain independent SNPs as IVs. In reverse and replicate MR analyses, the same criteria were adapted. A comprehensive summary of the selected IVs can be found in Table S2, Supplemental Digital Content, http://links.lww.com/MD/O500. Additionally, we performed MR Pleiotropy RESidual Sum and Outlier analysis to identify significant SNPs that might exhibit pleiotropic effects.^[39]^ Any significant outliers identified during this analysis were eliminated. To assess the strength of our MR approach, we employed the *F*-statistics = (Beta/Se)^2^. Beta represents the correlation coefficient between SNPs and exposure. If the *F*-statistics fell below 10, indicating insufficient strength, the corresponding SNP was not considered.^[40]^ The most influential genetic loci associated with these autoimmune conditions are predominantly located within the major histocompatibility complex (MHC), a region of paramount significance involved in adaptive immune responses. Therefore, we excluded variants within the MHC region, situated between base positions 24,000,000 to 35,000,000 on chromosome 6 (GRCh37), from our analysis due to their strong association with autoimmune diseases and the complex linkage disequilibrium structure within this region.^[41–43]^ This exclusion measure aimed to mitigate potential horizontal pleiotropy and uphold the core assumptions of the MR framework. Subsequently, we conducted the MR analysis using instruments unrelated to the MHC region with the intention of minimizing the influence of confounding factors. We also used Steiger filtering to ensure the directionality of the association.^[44]^

### 2.5. Statistical analysis

In this MR study, we primarily utilized the random effects inverse variance weighting method to investigate causal associations between autoimmune diseases and glioma. For cases where only 1 SNP was available, we employed the Wald ratio method. To address multiple comparisons, we implemented a Bonferroni correction with a threshold *P*-value of .05/75 (*P* < .0006, 25 × 3) and prioritized results for further analysis. Associations with *P* < .0006 were deemed significant, while associations with *P* ≥ .0006 and <.05 were considered suggestive. We used a fixed-effect method to combine the MR results obtained from the UKB and FinnGen consortiums. We conducted the entire analysis using R software (version 4.2.3, https://www.r-project.org/) along with “TwoSampleMR (version 0.5.6),” “mr.raps (version 0.4.1),” “MRPRESSO (version 1.0),” and “ggplot2 (version 3.4.0).”

### 2.6. Sensitivity analysis

In the reverse analysis for glioma, we applied the same screening criterion. The effects were estimated using multiple methods, including MR-Egger, weighted median, simple mode, and weighted mode. In the MR-Egger analysis, the introduction of an intercept term allowed for the evaluation of the Instrument Strength Independent of the Direct Effect assumption. A *P* value <0.05 indicated potential horizontal pleiotropy. The weighted median analysis corrected for the estimation of the causal effect when at least half of the IVs were invalid.^[44,45]^ Maximum likelihood estimation provided unbiased results under the assumptions of no heterogeneity and absence of horizontal pleiotropy.^[46]^ The global test in MR Pleiotropy RESidual Sum and Outlier was employed to assess overall horizontal pleiotropy and correct estimates by removing significant outliers.^[39]^ MR robust adjusted profile enhanced statistical power by accounting for weak instrumental variables.^[47]^ We also performed Cochran *Q* test to explore the heterogeneity among variant-specific estimates. In addition, leave-one-out analysis was conducted to verify the robustness of the conclusion.

## 3. Results

### 3.1. Causal effects of the genetically predicted autoimmune diseases on glioma and GBM in MR and replicate MR analysis

We did not collect satisfied SNP used as IVs for Behcet disease, idiopathic thrombocytopenic purpura, polymyalgia rheumatica, rheumatic fever, systemic sclerosis, Wegener granulomatosis, and adrenocortical insufficiency in UKB because of the threshold *P* value. In the FinnGen consortium, we did not identify IVs for Behcet disease, idiopathic thrombocytopenic purpura, rheumatic fever, systemic sclerosis, Wegener granulomatosis, and adrenocortical insufficiency. Collectively, our MR analysis included 18 exposures in UKB and 19 exposures in FinnGen. Table S2, Supplemental Digital Content, http://links.lww.com/MD/O500 and Table S3, Supplemental Digital Content, http://links.lww.com/MD/O501 provided a summary of SNPs utilized as IVs for exposure from UKB and FinnGen, respectively. None of these IVs had an *F*-statistic below the threshold of 10 in the strength of the MR analysis using *F*-statistics = (Bets/Se)^2^. SNPs in MR analysis were not weak IVs. The MR results were presented in Table S4, Supplemental Digital Content, http://links.lww.com/MD/O502.

**Figure 2.**
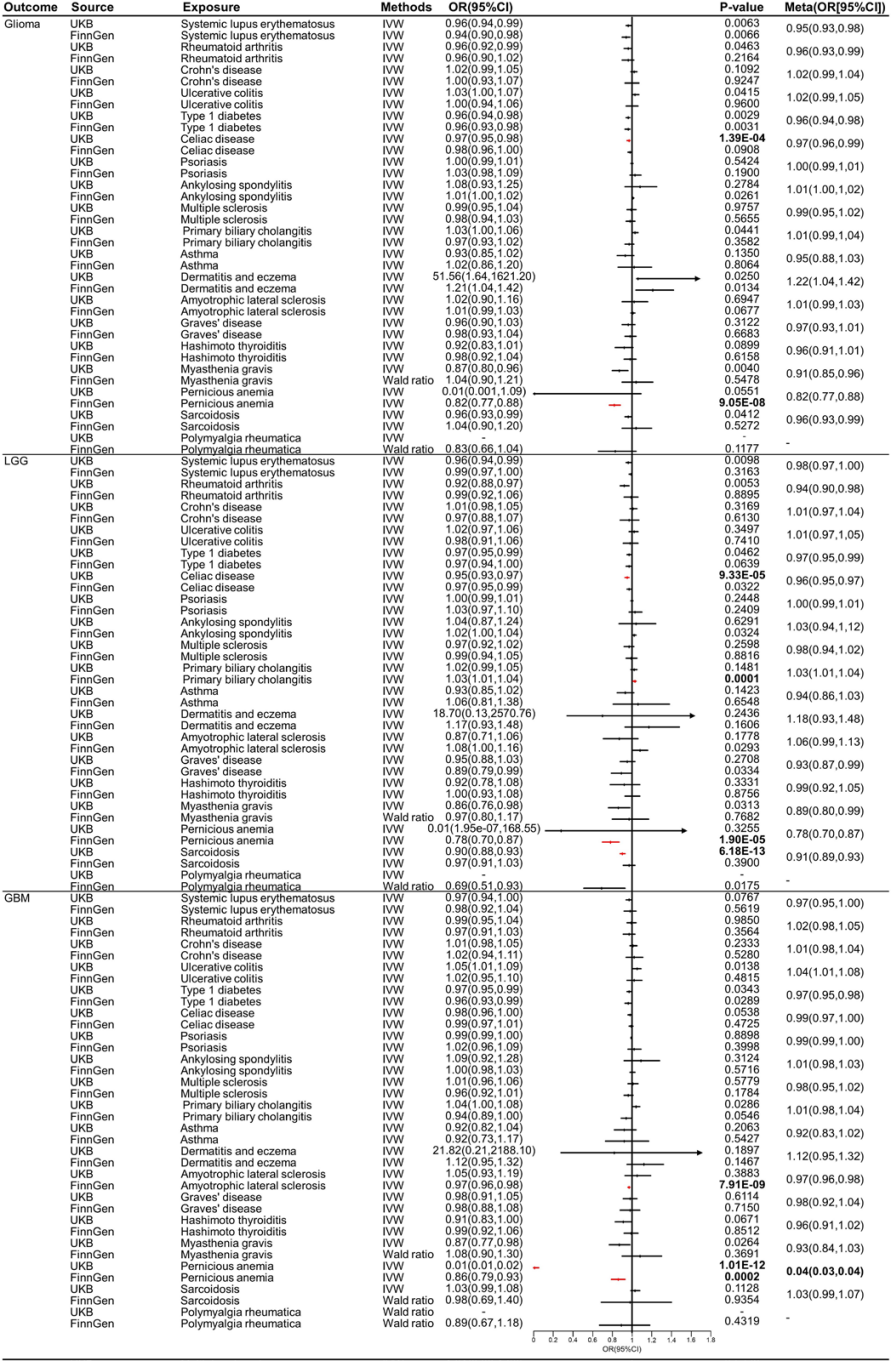
The causal effect of autoimmune diseases on glioma, LGG, and GBM. LGG = low-grade glioma, GBM = glioblastoma.

Based on the inverse variance weighting or Wald ratio method (Fig. 2), the results indicated that pernicious anemia might decrease the risk of GBM (UKB: odds ratio (OR) = 0.01, 95% confidence interval CI = 0.01–0.02, *P* = 1.01E‐12; FinnGen: OR = 0.86, 95% CI = 0.79–0.93, *P* = .0002; Meta: OR = 0.04, 95% CI = 0.03–0.04). The pernicious anemia had a protective role in the GBM development (OR < 1). The causal association between SLE (UKB: OR = 0.96, 95% CI = 0.94–0.99, *P* = .0063; FinnGen: OR = 0.94, 95% CI = 0.90–0.98, *P* = .0066; Meta: OR = 0.95, 95% CI = 0.93–0.98), T1D (UKB: OR = 0.96, 95% CI = 0.94–0.98, *P* = .0029; FinnGen: OR = 0.96, 95% CI = 0.93–0.98, *P* = .0031; Meta: OR = 0.96, 95% CI = 0.94–0.98), Eczema or dermatitis (UKB: OR = 51.56, 95% CI = 1.64–1621.20, *P* = .0250; FinnGen: OR = 1.21, 95% CI = 1.04–1.42, *P* = .0134; Meta: OR = 1.22, 95% CI = 1.04–1.42), and glioma, Celiac disease (UKB: OR = 0.95, 95% CI = 0.93–0.97, *P* = 9.33E‐05; FinnGen: OR = 0.97, 95% CI = 0.95–0.99, *P* = .0322; Meta: OR = 0.96, 95% CI = 0.95–0.97) and LGG, and T1D (UKB: OR = 0.97, 95% CI = 0.95–0.99, *P* = .0343; FinnGen: OR = 0.96, 95% CI = 0.93–0.99, *P* = .0289; Meta: OR = 0.97, 95% CI = 0.95–0.98) and GBM were suggestive (Figures S1, S5, S9, S13, S17, and S21, Supplemental Digital Content, http://links.lww.com/MD/O503). In contrast, we did not find causal associations between autoimmune diseases and glioma and LGG using UKB and FinnGen datasets in MR and replicate MR analyses (*P* > .05). Results in all funnel plots suggested no signs of heterogeneity (Figures S2, S6, S10, S14, S18, and S22, Supplemental Digital Content, http://links.lww.com/MD/O504). Sensitivity analyses did not find signs of pleiotropy (Table S4, Supplemental Digital Content, http://links.lww.com/MD/O502). Moreover, the results of Cochran *Q* test demonstrated no signs of heterogeneity. Estimates for every SNP are shown in Figures S3, S7, S11, S15, S19, and S23, Supplemental Digital Content, http://links.lww.com/MD/O505. Furthermore, results of the leave-one-out analyses suggested that no influential IVs were identified after removing any SNP in turn (Figures S4, S8, S12, S16, S20, and S24, Supplemental Digital Content, http://links.lww.com/MD/O506).

### 3.2. Causal effects of the genetically predicted glioma on autoimmune diseases in reverse MR and replicate reverse MR analysis

**Figure 3.**
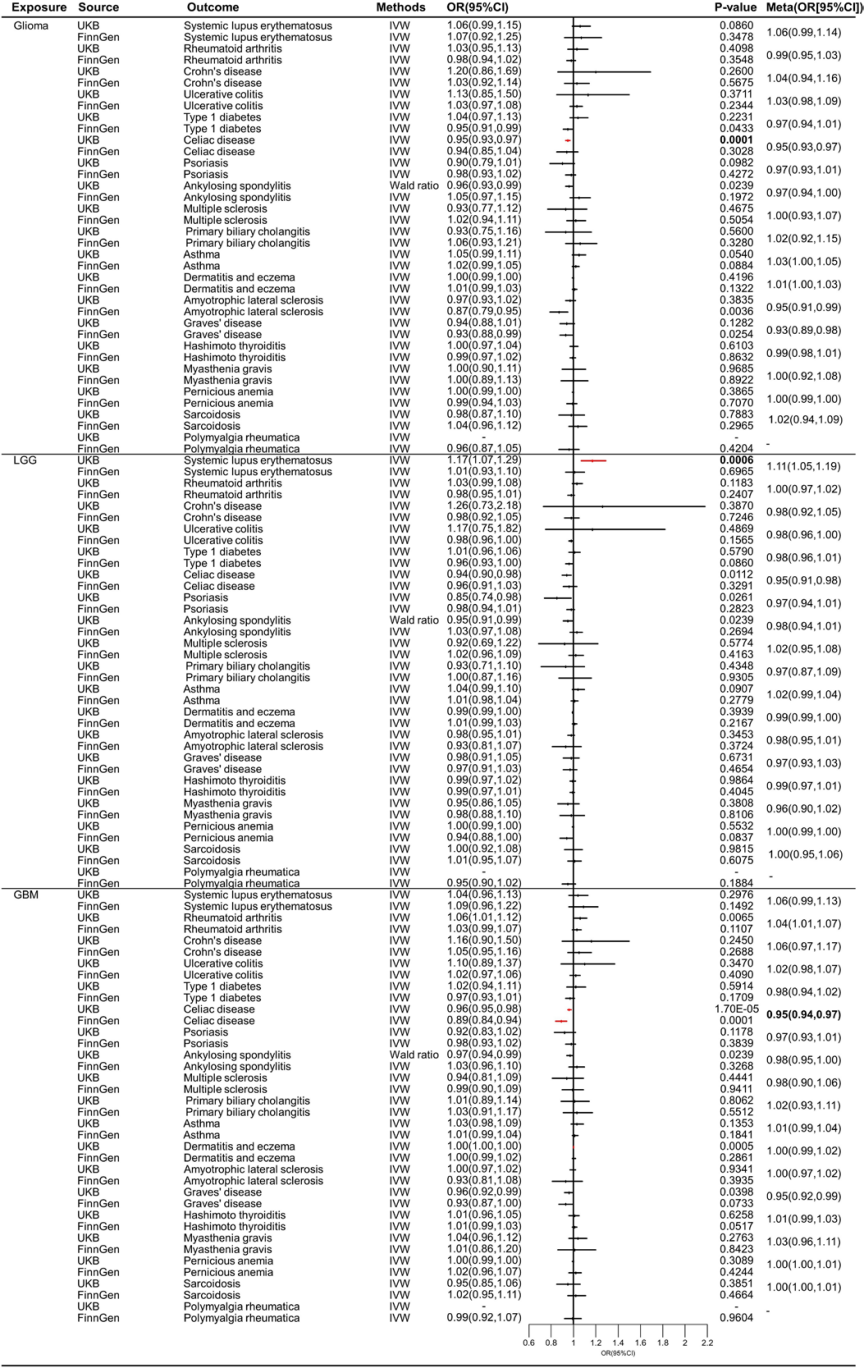
The causal effect of glioma, LGG, and GBM on autoimmune diseases. LGG = low-grade glioma, GBM = glioblastoma.

Table S5, Supplemental Digital Content, http://links.lww.com/MD/O507 provides a summary of SNPs utilized as IVs for glioma, LGG, GBM, and vestibular schwannoma, respectively. None of these IVs had an *F*-statistic below the threshold of 10. Table S6, Supplemental Digital Content, http://links.lww.com/MD/O508 shows the MR results of glioma on autoimmune diseases in reverse MR analysis. As shown in Figure 3, we found that GBM was causal with the decreased risk of celiac disease (UKB: OR = 0.96, 95% CI = 0.95–0.98, *P* = .0000; FinnGen: OR = 0.89, 95% CI = 0.84–0.94, *P* = .0001; Meta: OR = 0.95, 95% CI = 0.94–0.97) (Figures S25, S29, S33, S37, S41, and S45, Supplemental Digital Content, http://links.lww.com/MD/O509). GBM decreased the risk of Celiac disease (OR < 1). However, we did not identify causal association between autoimmune diseases and glioma and LGG in reverse MR and replicate reverse MR analysis (*P* > .05). Sensitivity analysis did not find the signs of pleiotropy and heterogeneity. Results in all funnel plots demonstrated no signs of heterogeneity (Figures S26, S30, S34, S38, S42, and S46, Supplemental Digital Content, http://links.lww.com/MD/O510). Causal estimates for each SNP were displayed in (Figures S27, S31, S35, S39, S43, and S47, Supplemental Digital Content, http://links.lww.com/MD/O511). The leave-one-out analyses did not identify influential IVs (Figures S28, S32, S36, S40, S44, and S48, Supplemental Digital Content, http://links.lww.com/MD/O512).

## 4. Discussion

To our knowledge, this study is the first to investigate the causal association between autoimmune diseases and glioma and its subtypes. Our findings suggest that genetically predicted pernicious anemia may reduce the risk of GBM, and conversely, GBM may lower the risk of celiac disease. Sensitivity analyses, including heterogeneity and pleiotropy analyses, as well as reverse analysis, confirmed the robustness of these results. These findings offer insights into the potential involvement of immunologic factors in the development of GBM.

The relationship between autoimmune diseases and glioma has been explored in a number of studies, but the conclusions remain inconsistent. Some studies have reported no causal association between autoimmune diseases and glioma.^[6,48]^ For instance, a nationwide Swedish Hospital Discharge Register study involving 402,462 patients found no significant relationship between autoimmune diseases and glioma, although the number of brain cancer cases was relatively small (880 brain cancers, including 317 gliomas). Similarly, a Clinical Practice Research Datalink GOLD study from the UK did not identify any altered association between a history of any autoimmune disease and glioma (OR 0.98, 95% CI 0.86–1.11). However, subgroup analyses among younger patients revealed a statistically significant increased risk of glioma in patients with inflammatory bowel disease (OR = 2.59, 95% CI 1.31–5.12).^[49]^ On the other hand, some studies have found an inverse association between autoimmune diseases and glioma.^[50–52]^ A meta-analysis involving 12 studies and 61,090 participants reported a reduced risk of glioma in individuals with allergic conditions (OR = 0.60, 95% CI = 0.52–0.69, *P* < .00). Subgroup analyses indicated a significant protective effect of asthma (OR = 0.70, 95% CI = 0.62–0.79, *P* < .001), eczema (OR = 0.69, 95% CI = 0.62–0.78, *P* < .001), and hay fever (OR = 0.78, 95% CI = 0.70–0.87, *P* < .001).^[50]^ However, this meta-analysis did not include analyses for glioma subtypes, and the study designs varied among the included studies. In a large hospital-based case-control study involving 782 brain cancers (including 489 gliomas) and 799 controls, researchers observed a significant inverse association between glioma and a history of allergies (OR = 0.67, 95% CI = 0.52–0.86) or autoimmune diseases (OR = 0.49, 95% CI = 0.35–0.69), suggesting that patients with allergies or autoimmune diseases may have a decreased risk of glioma. Subtype analysis was not performed in this study. Consistent with these findings, our study also identified a possible protective effect of pernicious anemia on GBM. However, we did not observe the inverse association of asthma, eczema, hay fever, and diabetes with glioma.

The brain is no longer considered an immune “privileged” site, as patients with immunocompromised status may develop infections, nervous system lymphoma, and even glioma.^[53]^ In many cases of glioma, there is a lack of an effective antitumor response, and evident immunocompromised status is often observed, particularly in GBM.^[54]^ Autoimmune diseases involve inappropriate immune responses, targeting either innocuous foreign substances or self-antigens. The potential protective role of a robust immune system against glioma remains uncertain. It is plausible that a common, yet undefined immune-related factor may predispose individuals to autoimmune diseases while simultaneously offering protection against glioma development. Recent research suggests that the immune system operates in close association with the nervous system, and this neural-immune interface plays a critical role in cancer progression and autoimmune disorders. In the case of GBM, neoplastic cells manipulate the neuro-immune axis to promote tumor growth, survival, and invasion.^[55,56]^

Pernicious anemia, characterized by vitamin B12 deficiency, has been linked to neuroblastoma cell behavior in previous in vitro studies, demonstrating reduced proliferation, enhanced differentiation, and induction of endoplasmic reticulum stress.^[57]^ The G2/M cell cycle arrest induced by vitamin B12 deficiency was the possible mechanisms of the inhibition of glioblastoma cells.^[58]^ In vitamin B12 deficiency, folate becomes “trapped” in its methylated form, disrupting cellular proliferation and potentially leading to DNA damage. A hallmark cellular consequence of cobalamin deficiency is the accumulation of homocysteine, alongside a reduction in the synthesis of methionine and S-adenosylmethionine, which can trigger oxidative stress, promote apoptosis, and lead to the homocysteinylation of functional proteins.^[59]^ It is widely recognized that rapidly proliferating cells in cancers have an increased demand for vitamin B12 compared to healthy cells, making them particularly vulnerable to deficiencies of these essential nutrients.^[60,61]^ Additionally, cobalamin transport antagonists have been explored as potential agents against GBM,^[58]^ suggesting a potential protective role of pernicious anemia in GBM development. Recent scientific literature has highlighted the biological interconnections between the gut and the brain. Celiac disease pathology involves the activation of CD4^+^ T-lymphocytes, B cell expansion, and CD8^+^ T-lymphocytes.^[62]^ Immunosuppression, both locally within tumors and systemically, is common in GBM, leading to dysfunction in tumor-induced T cells and NK cells, as well as expansion of regulatory T cells and myeloid-derived suppressor cells. These factors may contribute to the observed mitigative effect of GBM on celiac disease.

This is the first comprehensive MR study to explore causal association between 25 autoimmune diseases and glioma, LGG, and GBM from the genetic perspective, which provides valuable insights into the role of different autoimmune conditions in glioma development. Both discovery and replicate MR analyses supported these findings. Second, there is no overlap between exposure and outcome, which mitigates bias. The MR design circumvents confounding factors and reversed causality inherent in previous observational studies. Third, the MR results are validated through primary and replicate analyses, enhancing reliability. We comprehensively examined 25 autoimmune diseases. Fourth, we set the strict criterions to select genetic variants, including MHC exclusion and the removal of linkage disequilibrium, which are closely associated with exposures and avoid possible pleiotropy.

However, this study has its limitations. Primarily, the analysis is limited to populations of European ancestry, limiting the generalizability of the results to other ethnicities. There is a lack of GWAS datasets on Asian and African people, including 25 autoimmune diseases and glioma and its subtypes. Therefore, our results are mainly applied to Europeans. Additionally, the lack of autoimmune disease subtypes, including stratified risk factors related to disease duration, severity, treatment, and complications, precludes further analysis. Third, the potential residual confounding factors from unmeasured genetic or environment in both the exposure and outcome variables may influence the reliability of results. Future studies, replication in other ancestries, more rigorous clinical study design, and large studies with more samples, should be conducted to verify the conclusions and elucidate the possible mechanisms.

## 5. Conclusion

In summary, genetically predicted pernicious anemia may decrease the risk of GBM, while GBM may decrease the risk of celiac disease. These findings underscore the importance of autoimmune status in GBM risk assessment and highlight the need for future studies to explore underlying mechanisms. Understanding these associations could inform personalized medicine approaches, such as tailored surveillance strategies for high-risk individuals and potential therapeutic interventions targeting immune-related pathways. Future studies are needed to validate their association, clarify the underlying mechanisms, and assess their translational potential in clinical settings.

## Acknowledgments

We thank the participants and working staff for their contribution to the study in UKB and FinnGen consortium.

## Author contributions

**Conceptualization:** Ni Deng.

**Data curation:** Ni Deng.

**Formal analysis:** Ni Deng, Chao You.

**Investigation:** Ni Deng, Chao You, Songping Zheng.

**Methodology:** Ni Deng, Rafeq Agila, Chao You, Songping Zheng.

**Project administration:** Songping Zheng.

**Resources:** Ni Deng, Rafeq Agila, Qiang He.

**Software:** Ni Deng, Rafeq Agila.

**Supervision:** Qiang He.

**Validation:** Qiang He, Songping Zheng.

**Visualization:** Ni Deng, Chao You, Songping Zheng.

**Writing – original draft:** Ni Deng.

**Writing – review & editing:** Songping Zheng.

## Supplementary Material

## References

[1] Vincent A Bien CG Irani SR Waters P. Autoantibodies associated with diseases of the CNS: new developments and future challenges. Lancet Neurol. 2011;10:759–72. 21777830 10.1016/S1474-4422(11)70096-5

[2] Wraith DC Nicholson LB. The adaptive immune system in diseases of the central nervous system. J Clin Invest. 2012;122:1172–9. 22466659 10.1172/JCI58648 PMC3314451

[3] Bernatsky S Ramsey-Goldman R Clarke A. Malignancy and autoimmunity. Curr Opin Rheumatol. 2006;18:129–34. 16462517 10.1097/01.bor.0000209423.39033.94

[4] Goldin LR Landgren O. Autoimmunity and lymphomagenesis. Int J Cancer. 2009;124:1497–502. 19089924 10.1002/ijc.24141 PMC2666348

[5] Linos E Raine T Alonso A Michaud D. Atopy and risk of brain tumors: a meta-analysis. J Natl Cancer Inst. 2007;99:1544–50. 17925535 10.1093/jnci/djm170

[6] Hemminki K Liu X Forsti A Ji J Sundquist J Sundquist K. Subsequent brain tumors in patients with autoimmune disease. Neuro Oncol. 2013;15:1142–50. 23757294 10.1093/neuonc/not070 PMC3748918

[7] Hemminki K Forsti A Fallah M Sundquist J Sundquist K Ji J. Risk of cancer in patients with medically diagnosed hay fever or allergic rhinitis. Int J Cancer. 2014;135:2397–403. 24692097 10.1002/ijc.28873

[8] Borghaei H Paz-Ares L Horn L. Nivolumab versus docetaxel in advanced nonsquamous non-small-cell lung cancer. N Engl J Med. 2015;373:1627–39. 26412456 10.1056/NEJMoa1507643 PMC5705936

[9] Robert C Schachter J Long GV. Pembrolizumab versus ipilimumab in advanced melanoma. N Engl J Med. 2015;372:2521–32. 25891173 10.1056/NEJMoa1503093

[10] Touat M Idbaih A Sanson M Ligon KL. Glioblastoma targeted therapy: updated approaches from recent biological insights. Ann Oncol. 2017;28:1457–72. 28863449 10.1093/annonc/mdx106 PMC5834086

[11] Eriksson NE Holmen A Hogstedt B Mikoczy Z Hagmar L. A prospective study of cancer incidence in a cohort examined for allergy. Allergy. 1995;50:718–22. 8546265 10.1111/j.1398-9995.1995.tb01212.x

[12] Emdin CA Khera AV Kathiresan S. Mendelian randomization. JAMA. 2017;318:1925–6. 29164242 10.1001/jama.2017.17219

[13] Smith GD Ebrahim S. “Mendelian randomization”: can genetic epidemiology contribute to understanding environmental determinants of disease? Int J Epidemiol. 2003;32:1–22. 12689998 10.1093/ije/dyg070

[14] He Q Wang W Li H. Genetic insights into the risk of metabolic syndrome and its components on stroke and its subtypes: bidirectional Mendelian randomization. J Cereb Blood Flow Metab. 2023;43(2 Suppl):126–37. 37198928 10.1177/0271678X231169838 PMC10638990

[15] Burgess S Butterworth A Thompson SG. Mendelian randomization analysis with multiple genetic variants using summarized data. Genet Epidemiol. 2013;37:658–65. 24114802 10.1002/gepi.21758 PMC4377079

[16] Skrivankova VW Richmond RC Woolf BR. Strengthening the reporting of observational studies in epidemiology using Mendelian randomization: the STROBE-MR statement. JAMA. 2021;326:1614–21. 34698778 10.1001/jama.2021.18236

[17] Skrivankova VW Richmond RC Woolf BR. Strengthening the reporting of observational studies in epidemiology using Mendelian randomisation (STROBE-MR): explanation and elaboration. BMJ. 2021;375:n2233. 34702754 10.1136/bmj.n2233 PMC8546498

[18] Hemani G Zheng J Elsworth B. The MR-Base platform supports systematic causal inference across the human phenome. Elife. 2018;7:e34408. 29846171 10.7554/eLife.34408 PMC5976434

[19] Ha E Bae SC Kim K. Large-scale meta-analysis across East Asian and European populations updated genetic architecture and variant-driven biology of rheumatoid arthritis, identifying 11 novel susceptibility loci. Ann Rheum Dis. 2021;80:558–65. 33310728 10.1136/annrheumdis-2020-219065 PMC8053349

[20] Liu JZ Van Sommeren S Huang H. Association analyses identify 38 susceptibility loci for inflammatory bowel disease and highlight shared genetic risk across populations. Nat Genet. 2015;47:979–86. 26192919 10.1038/ng.3359 PMC4881818

[21] Bentham J Morris DL Graham DSC. Genetic association analyses implicate aberrant regulation of innate and adaptive immunity genes in the pathogenesis of systemic lupus erythematosus. Nat Genet. 2015;47:1457–64. 26502338 10.1038/ng.3434 PMC4668589

[22] Forgetta V Manousaki D Istomine R. Rare genetic variants of large effect influence risk of type 1 diabetes. Diabetes. 2020;69:784–95. 32005708 10.2337/db19-0831 PMC7085253

[23] Trynka G Hunt KA Bockett NA. Dense genotyping identifies and localizes multiple common and rare variant association signals in celiac disease. Nat Genet. 2011;43:1193–201. 22057235 10.1038/ng.998 PMC3242065

[24] Tsoi LC Spain SL Knight J. Identification of 15 new psoriasis susceptibility loci highlights the role of innate immunity. Nat Genet. 2012;44:1341–8. 23143594 10.1038/ng.2467 PMC3510312

[25] Cortes A Hadler J Pointon JP. Identification of multiple risk variants for ankylosing spondylitis through high-density genotyping of immune-related loci. Nat Genet. 2013;45:730–8. 23749187 10.1038/ng.2667 PMC3757343

[26] Beecham AH Patsopoulos NA Xifara DK. Analysis of immune-related loci identifies 48 new susceptibility variants for multiple sclerosis. Nat Genet. 2013;45:1353–60. 24076602 10.1038/ng.2770 PMC3832895

[27] Cordell HJ Han Y Mells GF. International genome-wide meta-analysis identifies new primary biliary cirrhosis risk loci and targetable pathogenic pathways. Nat Commun. 2015;6:8019. 26394269 10.1038/ncomms9019 PMC4580981

[28] Demenais F Margaritte-Jeannin P Barnes KC. Multiancestry association study identifies new asthma risk loci that colocalize with immune-cell enhancer marks. Nat Genet. 2018;50:42–53. 29273806 10.1038/s41588-017-0014-7 PMC5901974

[29] Donertas HM Fabian DK Valenzuela MF Partridge L Thornton JM. Common genetic associations between age-related diseases. Nat Aging. 2021;1:400–12. 33959723 10.1038/s43587-021-00051-5 PMC7610725

[30] Van Rheenen W Van Der Spek RA Bakker MK. Common and rare variant association analyses in amyotrophic lateral sclerosis identify 15 risk loci with distinct genetic architectures and neuron-specific biology. Nat Genet. 2021;53:1636–48. 34873335 10.1038/s41588-021-00973-1 PMC8648564

[31] Sakaue S Kanai M Tanigawa Y. A cross-population atlas of genetic associations for 220 human phenotypes. Nat Genet. 2021;53:1415–24. 34594039 10.1038/s41588-021-00931-x PMC12208603

[32] Chia R Saez-Atienzar S Murphy N. Identification of genetic risk loci and prioritization of genes and pathways for myasthenia gravis: a genome-wide association study. Proc Natl Acad Sci U S A. 2022;119:e2108672119. 35074870 10.1073/pnas.2108672119 PMC8812681

[33] Glanville KP Coleman JRI O’reilly PF Galloway J Lewis CM. Investigating pleiotropy between depression and autoimmune diseases using the UK biobank. Biol Psychiatry Glob Open Sci. 2021;1:48–58. 34278373 10.1016/j.bpsgos.2021.03.002 PMC8262258

[34] Backman JD Li AH Marcketta A. Exome sequencing and analysis of 454,787 UK Biobank participants. Nature. 2021;599:628–34. 34662886 10.1038/s41586-021-04103-z PMC8596853

[35] Lopez-Isac E Acosta-Herrera M Kerick M. GWAS for systemic sclerosis identifies multiple risk loci and highlights fibrotic and vasculopathy pathways. Nat Commun. 2019;10:4955. 31672989 10.1038/s41467-019-12760-y PMC6823490

[36] Jiang L Zheng Z Fang H Yang J. A generalized linear mixed model association tool for biobank-scale data. Nat Genet. 2021;53:1616–21. 34737426 10.1038/s41588-021-00954-4

[37] Kurki MI Karjalainen J Palta P. FinnGen provides genetic insights from a well-phenotyped isolated population. Nature. 2023;613:508–18. 36653562 10.1038/s41586-022-05473-8 PMC9849126

[38] Melin BS Barnholtz-Sloan JS Wrensch MR. Genome-wide association study of glioma subtypes identifies specific differences in genetic susceptibility to glioblastoma and non-glioblastoma tumors. Nat Genet. 2017;49:789–94. 28346443 10.1038/ng.3823 PMC5558246

[39] Verbanck M Chen CY Neale B Do R. Detection of widespread horizontal pleiotropy in causal relationships inferred from Mendelian randomization between complex traits and diseases. Nat Genet. 2018;50:693–8. 29686387 10.1038/s41588-018-0099-7 PMC6083837

[40] Chen L Yang H Li H He C Yang L Lv G. Insights into modifiable risk factors of cholelithiasis: a Mendelian randomization study. Hepatology. 2022;75:785–96. 34624136 10.1002/hep.32183 PMC9300195

[41] Ye CJ Kong LJ Wang YY. Mendelian randomization evidence for the causal effects of socio-economic inequality on human longevity among Europeans. Nat Hum Behav. 2023;7:1357–70. 37386110 10.1038/s41562-023-01646-1

[42] Wang L Wang FS Gershwin ME. Human autoimmune diseases: a comprehensive update. J Intern Med. 2015;278:369–95. 26212387 10.1111/joim.12395

[43] Dendrou CA Petersen J Rossjohn J Fugger L. HLA variation and disease. Nat Rev Immunol. 2018;18:325–39. 29292391 10.1038/nri.2017.143

[44] Hartwig FP Davey Smith G Bowden J. Robust inference in summary data Mendelian randomization via the zero modal pleiotropy assumption. Int J Epidemiol. 2017;46:1985–98. 29040600 10.1093/ije/dyx102 PMC5837715

[45] Bowden J Davey Smith G Haycock PC. Consistent estimation in Mendelian randomization with some invalid instruments using a weighted median estimator. Genet Epidemiol. 2016;40:304–14. 27061298 10.1002/gepi.21965 PMC4849733

[46] Pierce BL Burgess S. Efficient design for Mendelian randomization studies: subsample and 2-sample instrumental variable estimators. Am J Epidemiol. 2013;178:1177–84. 23863760 10.1093/aje/kwt084 PMC3783091

[47] Zhao Q Chen Y Wang J Small DS. Powerful three-sample genome-wide design and robust statistical inference in summary-data Mendelian randomization. Int J Epidemiol. 2019;48:1478–92. 31298269 10.1093/ije/dyz142

[48] Mills PK Preston-Martin S Annegers JF Beeson WL Phillips RL Fraser GE. Risk factors for tumors of the brain and cranial meninges in seventh-day adventists. Neuroepidemiology. 1989;8:266–75. 2812186 10.1159/000110193

[49] Anssar TM Leitzmann MF Linker RA. Autoimmune diseases and immunosuppressive therapy in relation to the risk of glioma. Cancer Med. 2020;9:1263–75. 31821741 10.1002/cam4.2767 PMC6997055

[50] Chen C Xu T Chen J. Allergy and risk of glioma: a meta-analysis. Eur J Neurol. 2011;18:387–95. 20722711 10.1111/j.1468-1331.2010.03187.x

[51] Cicuttini FM Hurley SF Forbes A. Association of adult glioma with medical conditions, family and reproductive history. Int J Cancer. 1997;71:203–7. 9139843 10.1002/(sici)1097-0215(19970410)71:2<203::aid-ijc13>3.0.co;2-i

[52] Schlehofer B Blettner M Preston-Martin S. Role of medical history in brain tumour development. Results from the international adult brain tumour study. Int J Cancer. 1999;82:155–60. 10389745 10.1002/(sici)1097-0215(19990719)82:2<155::aid-ijc1>3.0.co;2-p

[53] Brenner AV Linet MS Fine HA. History of allergies and autoimmune diseases and risk of brain tumors in adults. Int J Cancer. 2002;99:252–9. 11979441 10.1002/ijc.10320

[54] Sattiraju A Kang S Giotti B. Hypoxic niches attract and sequester tumor-associated macrophages and cytotoxic T cells and reprogram them for immunosuppression. Immunity. 2023;56:1825–43.e6. 37451265 10.1016/j.immuni.2023.06.017 PMC10527169

[55] Wang D Ayers MM Catmull DV Hazelwood LJ Bernard CCA Orian JM. Astrocyte-associated axonal damage in pre-onset stages of experimental autoimmune encephalomyelitis. Glia. 2005;51:235–40. 15812814 10.1002/glia.20199

[56] Mohme M Neidert MC. Tumor-specific T cell activation in malignant brain tumors. Front Immunol. 2020;11:205. 32117316 10.3389/fimmu.2020.00205 PMC7031483

[57] Ghemrawi R Pooya S Lorentz S. Decreased vitamin B12 availability induces ER stress through impaired SIRT1-deacetylation of HSF1. Cell Death Dis. 2013;4:e553. 23519122 10.1038/cddis.2013.69 PMC3615730

[58] Rzepka Z Rok J Maszczyk M. Response of human glioblastoma cells to vitamin B12 deficiency: a study using the non-toxic cobalamin antagonist. Biology (Basel). 2021;10:69. 33478021 10.3390/biology10010069 PMC7835758

[59] Green R Allen LH Bjorke-Monsen AL. Vitamin B(12) deficiency. Nat Rev Dis Primers. 2017;3:17040. 28660890 10.1038/nrdp.2017.40

[60] Zelder F Sonnay M Prieto L. Antivitamins for medicinal applications. ChemBioChem. 2015;16:1264–78. 26013037 10.1002/cbic.201500072

[61] Palmer AM Kamynina E Field MS Stover PJ. Folate rescues vitamin B(12) depletion-induced inhibition of nuclear thymidylate biosynthesis and genome instability. Proc Natl Acad Sci U S A. 2017;114:E4095–102. 28461497 10.1073/pnas.1619582114 PMC5441772

[62] Grabowski MM Sankey EW Ryan KJ. Immune suppression in gliomas. J Neurooncol. 2021;151:3–12. 32542437 10.1007/s11060-020-03483-y PMC7843555

