# Supplementary material for: Comprehensive causal analysis between autoimmune diseases and glioma: A Mendelian randomization study
Source: Medicine (Baltimore). 2025 Mar 7;104(10):e41815. doi: 10.1097/MD.0000000000041815 (PMC11902947; doi:10.1097/MD.0000000000041815)

Figure S2 The funnel plots of the association between genetically predicted autoimmune diseases from UKB and glioma in the MR analysis. SLE, Systemic lupus erythematosus; MR, Mendelian randomization; PBC, Primary biliary cholangitis; ALS, Amyotrophic lateral sclerosis

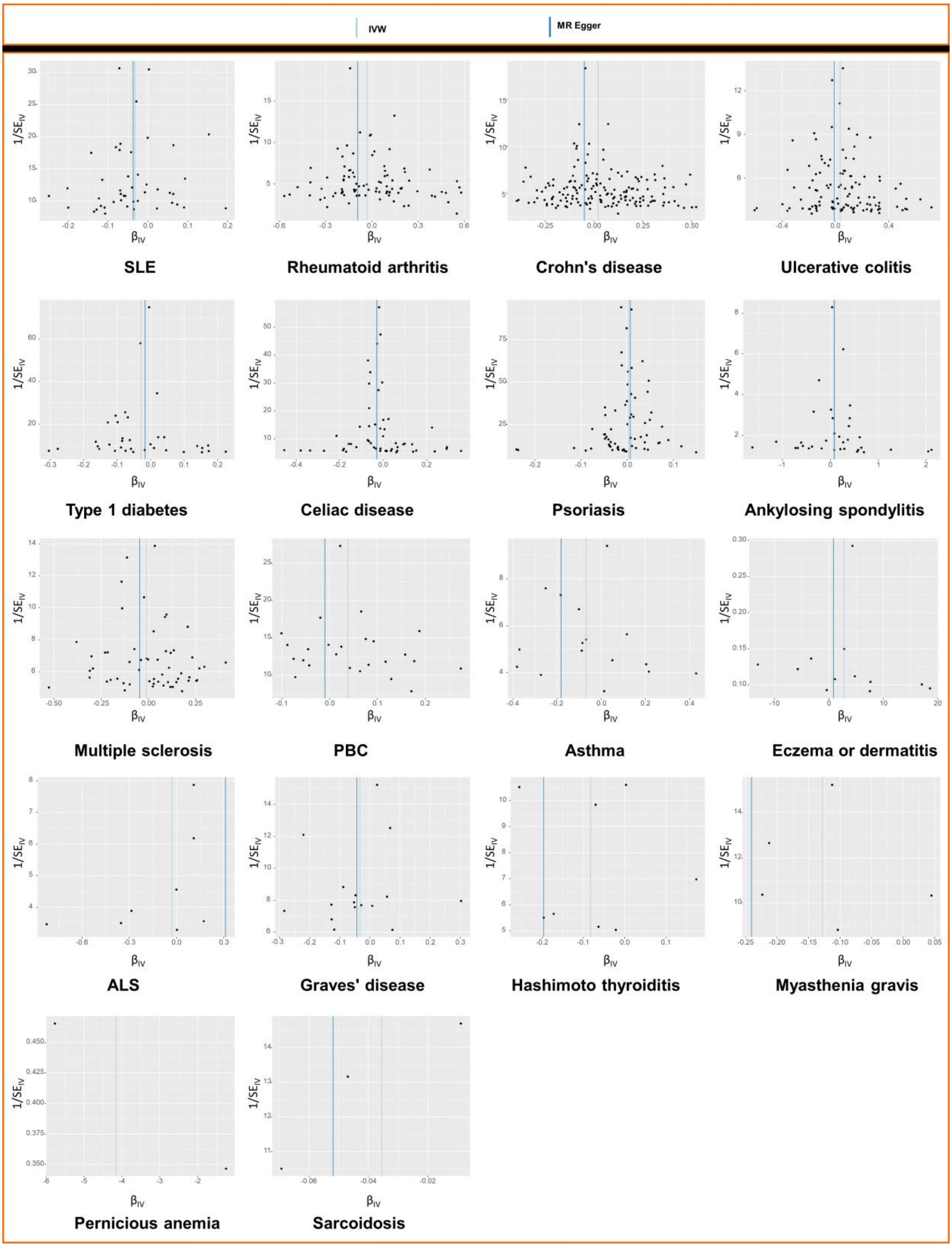

Figure S6 The funnel plots of the association between genetically predicted autoimmune diseases from UKB and LGG in the MR analysis. SLE, Systemic lupus erythematosus; MR, Mendelian randomization; PBC, Primary biliary cholangitis; ALS, Amyotrophic lateral sclerosis; LGG, lower-grade glioma

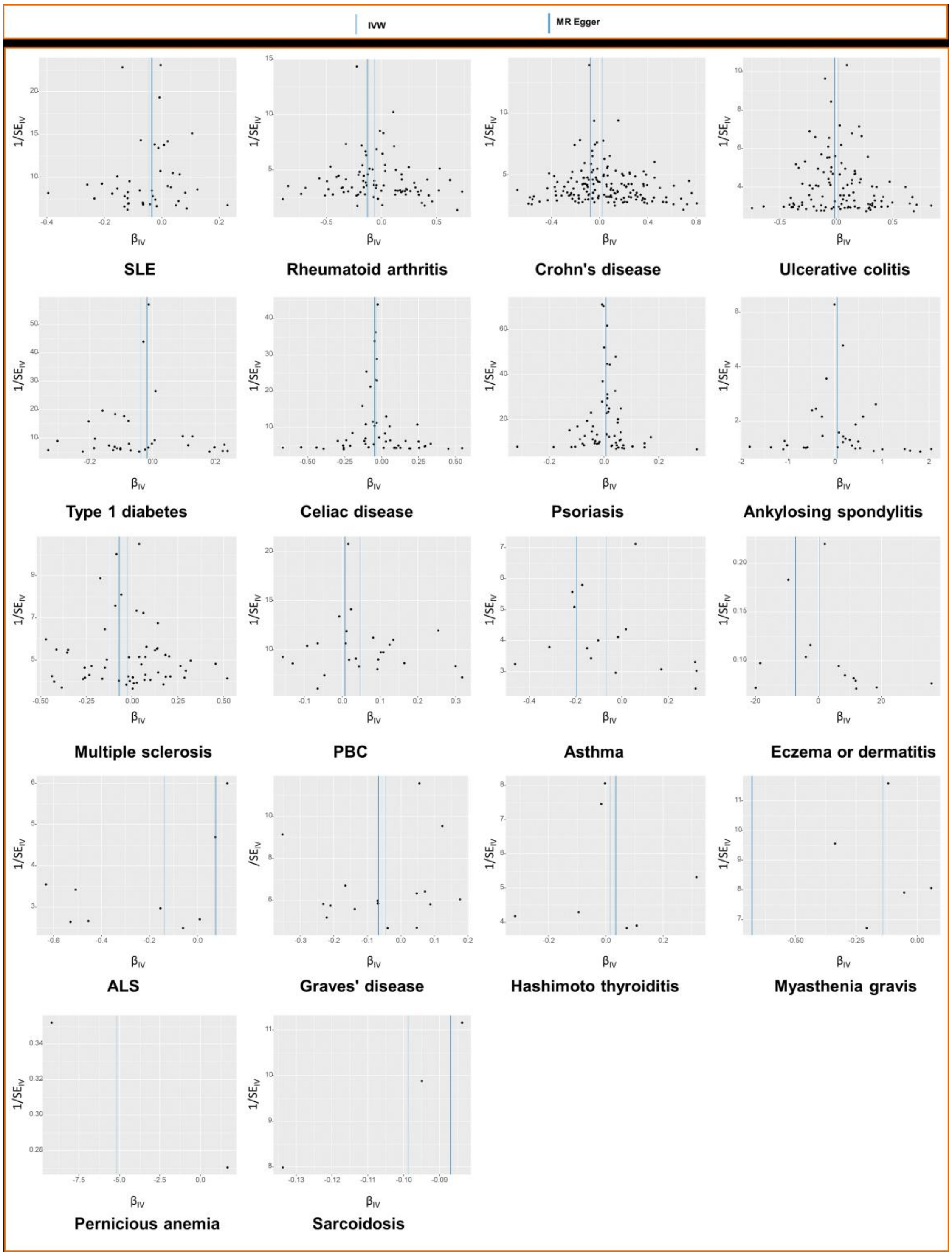

Figure S10 The funnel plots of the association between genetically predicted autoimmune diseases from UKB and GBM in the MR analysis. SLE, Systemic lupus erythematosus; MR, Mendelian randomization; GBM, glioblastoma; ALS, Amyotrophic lateral sclerosis

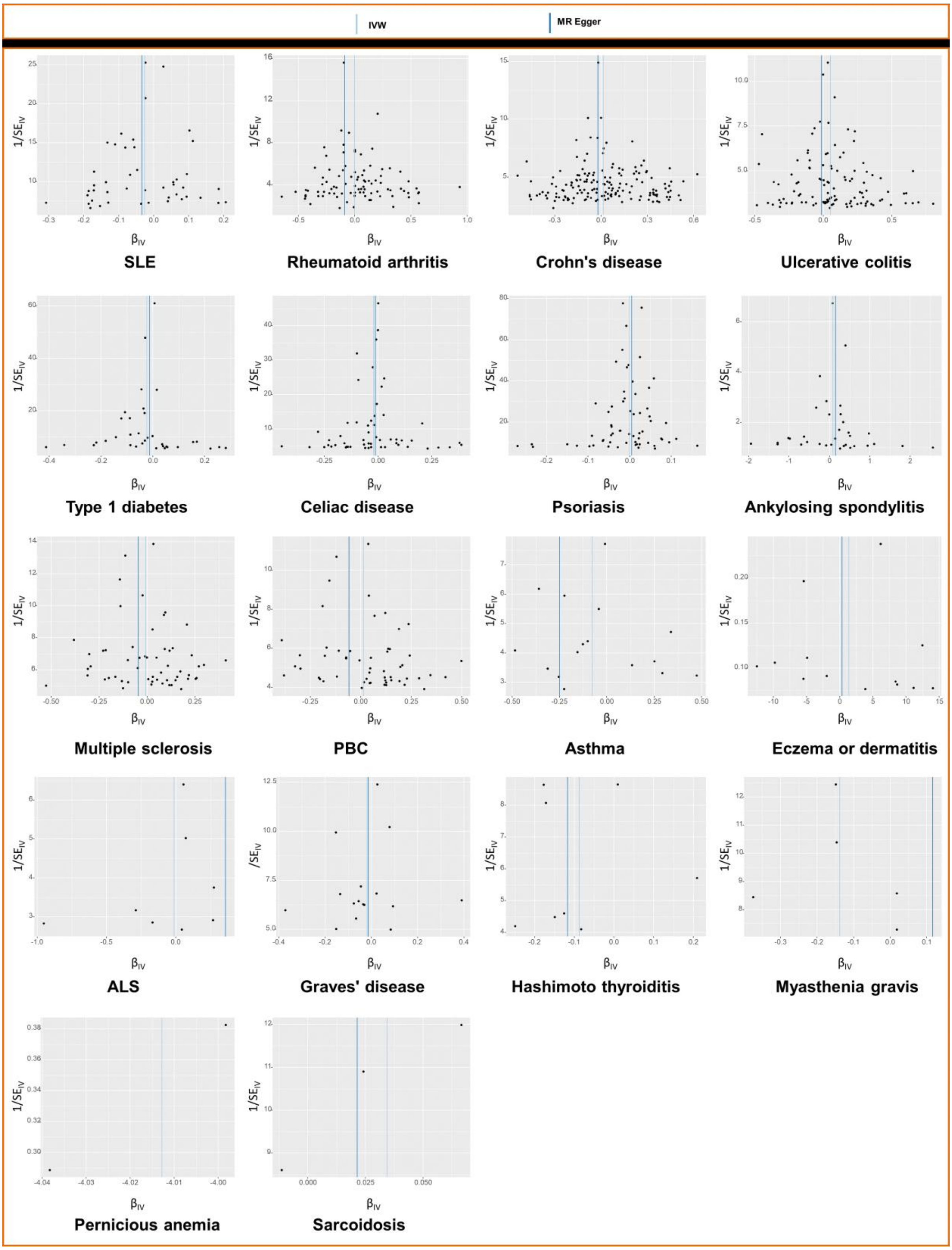

Figure S14 The funnel plots of the association between genetically predicted autoimmune diseases from FinnGen and glioma in the MR analysis. SLE, Systemic lupus erythematosus; MR, Mendelian randomization; PBC, Primary biliary cholangitis; ALS, Amyotrophic lateral sclerosis

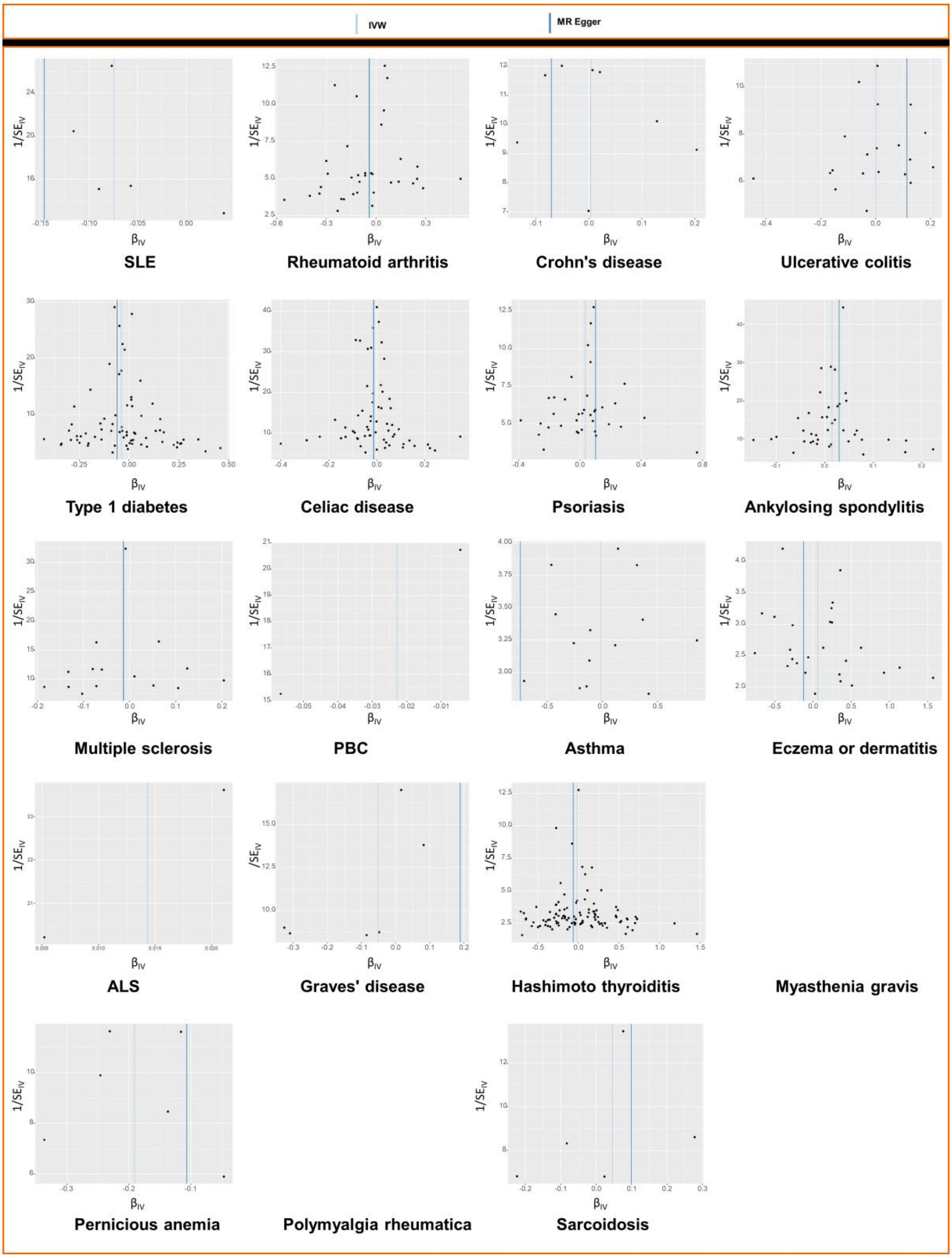

Figure S18 The funnel plots of the association between genetically predicted autoimmune diseases from FinnGen and LGG in the MR analysis. SLE, Systemic lupus erythematosus; MR, Mendelian randomization; PBC, Primary biliary cholangitis; ALS, Amyotrophic lateral sclerosis; LGG, lower-grade glioma

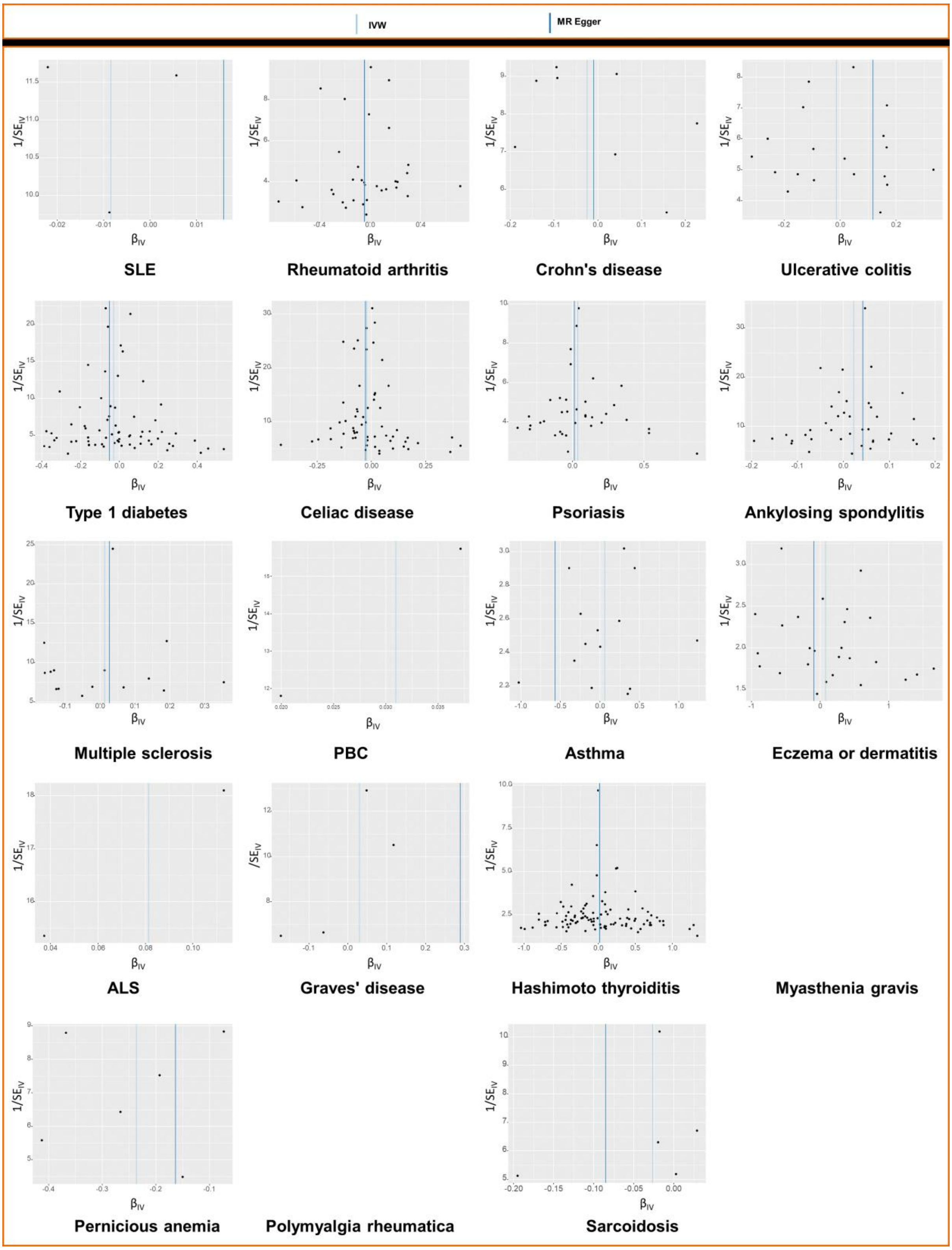

Figure S22 The funnel plots of the association between genetically predicted autoimmune diseases from FinnGen and GBM in the MR analysis. SLE, Systemic lupus erythematosus; MR, Mendelian randomization; PBC, Primary biliary cholangitis; ALS, Amyotrophic lateral sclerosis; GBM, glioblastoma

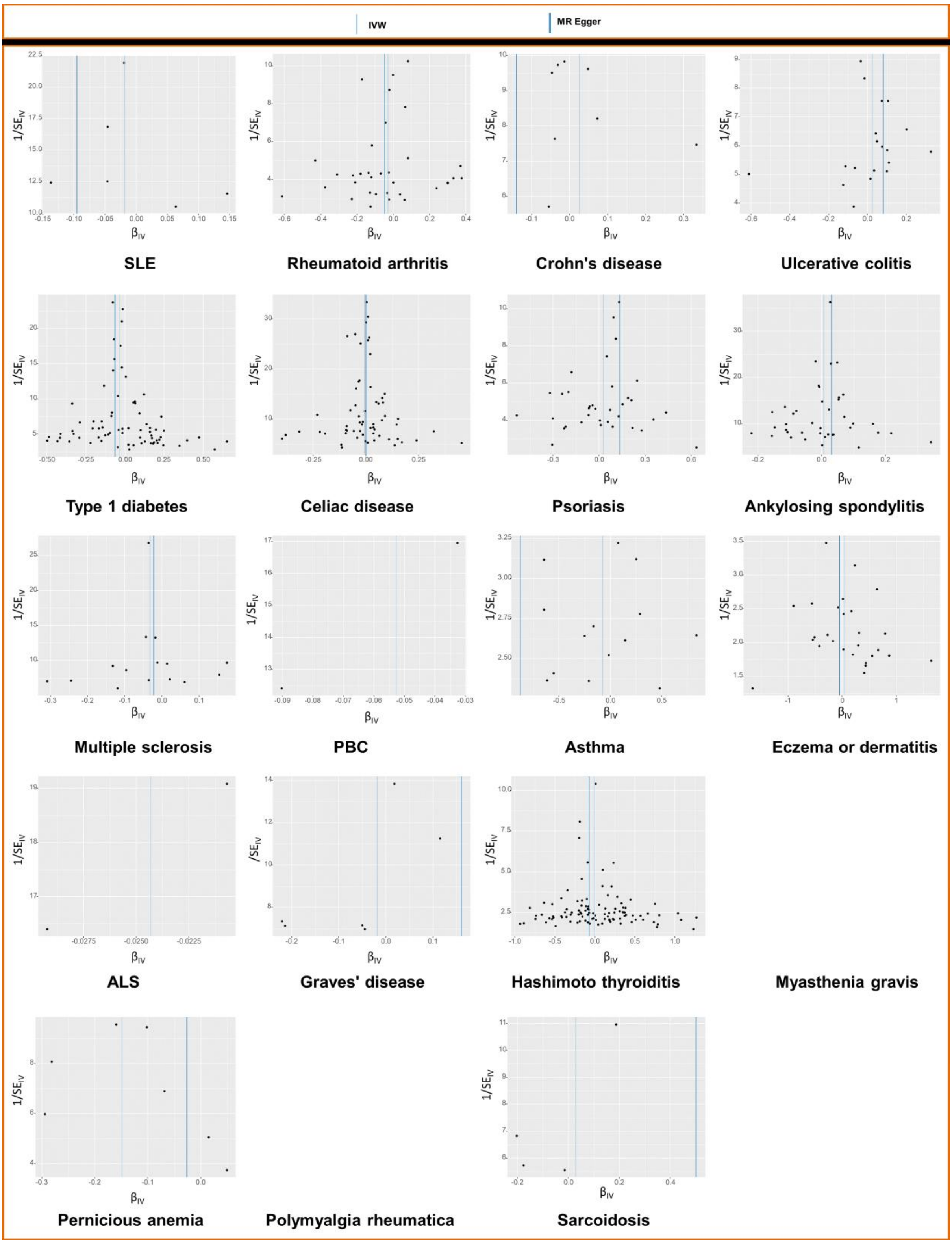

Supplement: Supplementary file 6 [file medi-104-e41815-s006.pdf]
