# Supplementary material for: Comprehensive causal analysis between autoimmune diseases and glioma: A Mendelian randomization study
Source: Medicine (Baltimore). 2025 Mar 7;104(10):e41815. doi: 10.1097/MD.0000000000041815 (PMC11902947; doi:10.1097/MD.0000000000041815)

**Figure S3** The frost plots of the association between genetically predicted autoimmune diseases from UKB and glioma in the MR analysis. SLE, Systemic lupus erythematosus; MR, Mendelian randomization; PBC, Primary biliary cholangitis; ALS, Amyotrophic lateral sclerosis


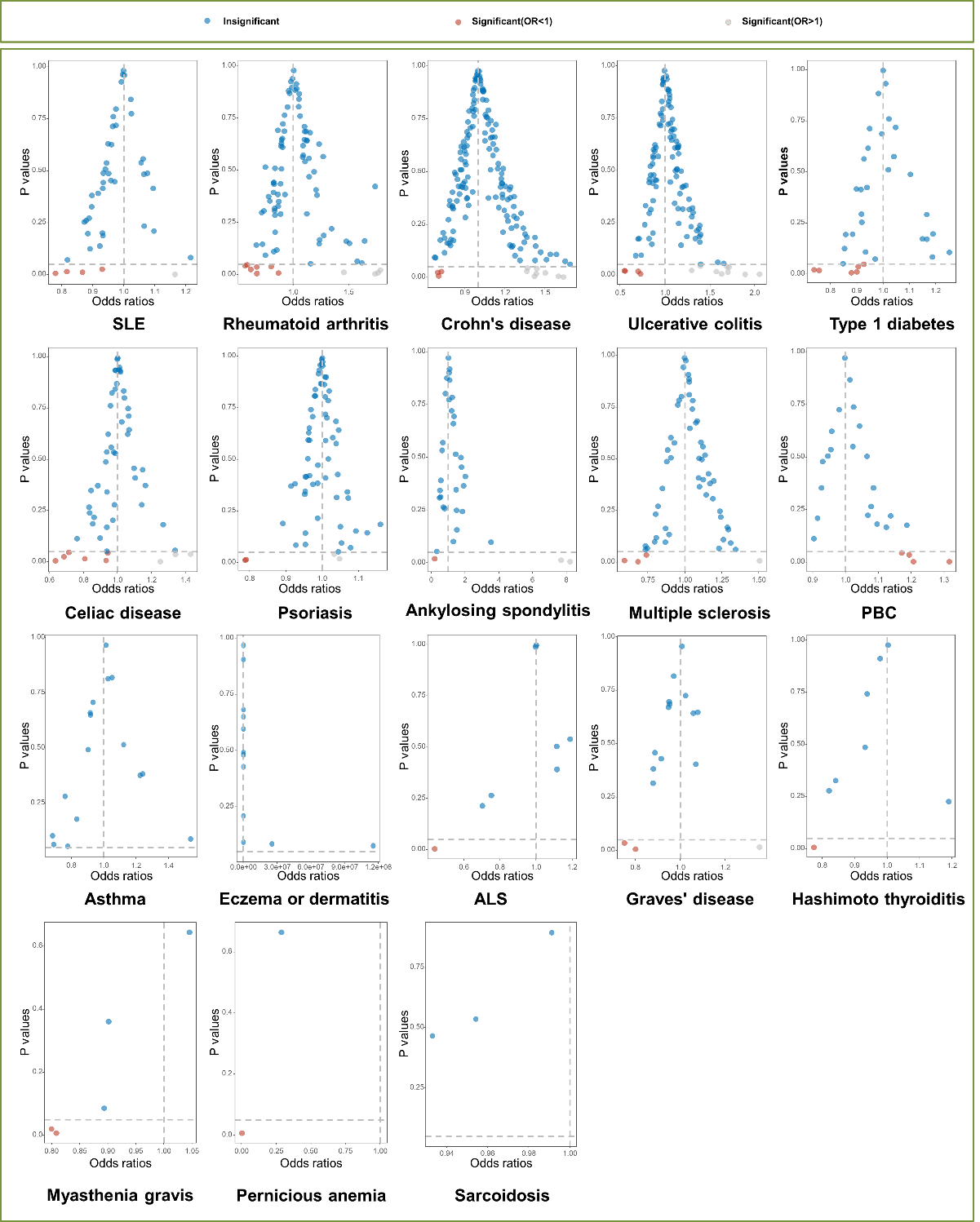


**Figure S7** The frost plots of the association between genetically predicted autoimmune diseases from UKB and LGG in the MR analysis. SLE, Systemic lupus erythematosus; MR, Mendelian randomization; PBC, Primary biliary cholangitis; ALS, Amyotrophic lateral sclerosis; LGG, lower-grade glioma


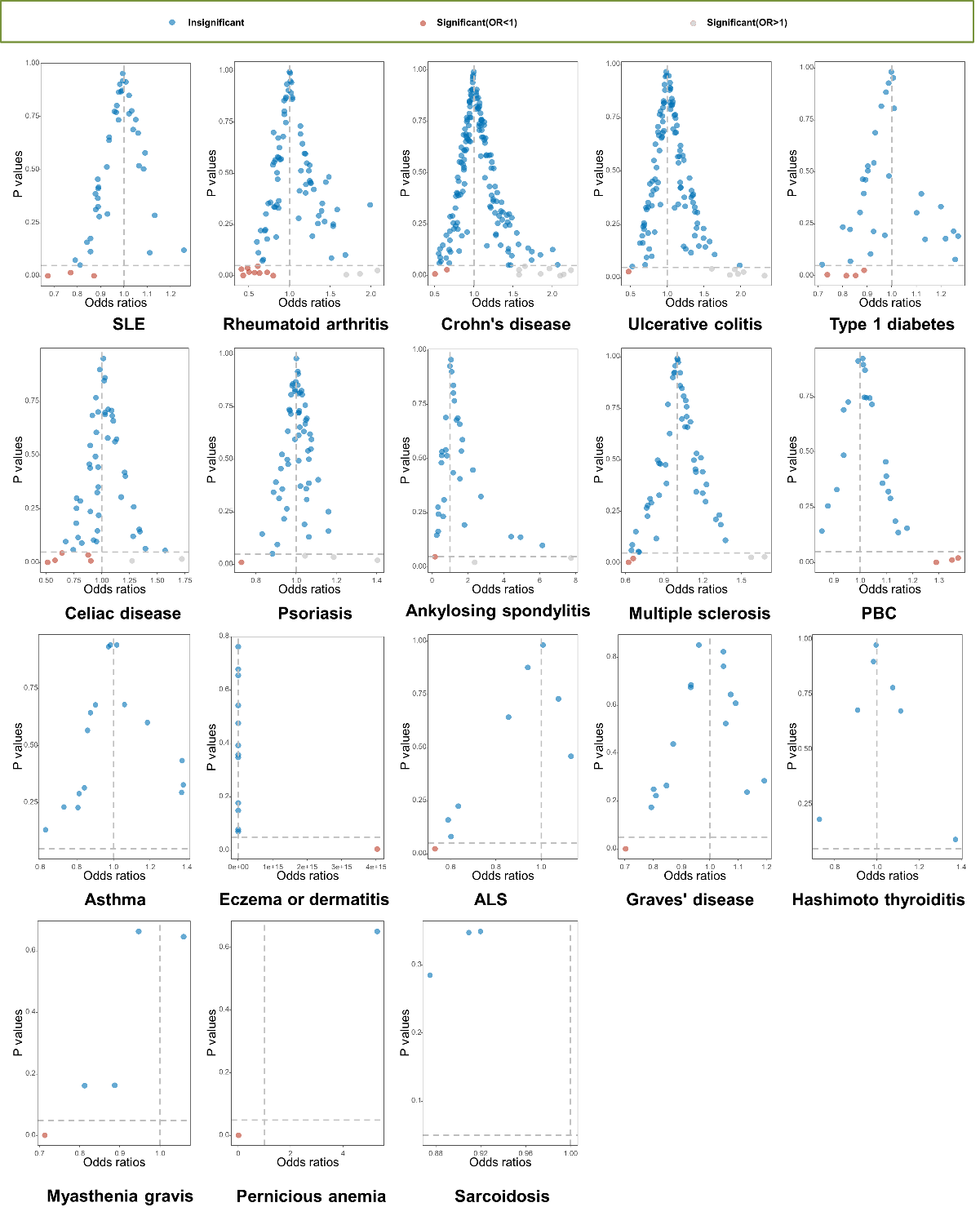


**Figure S11** The frost plots of the association between genetically predicted autoimmune diseases from UKB and GBM in the MR analysis. SLE, Systemic lupus erythematosus; MR, Mendelian randomization; GBM, glioblastoma; ALS, Amyotrophic lateral sclerosis


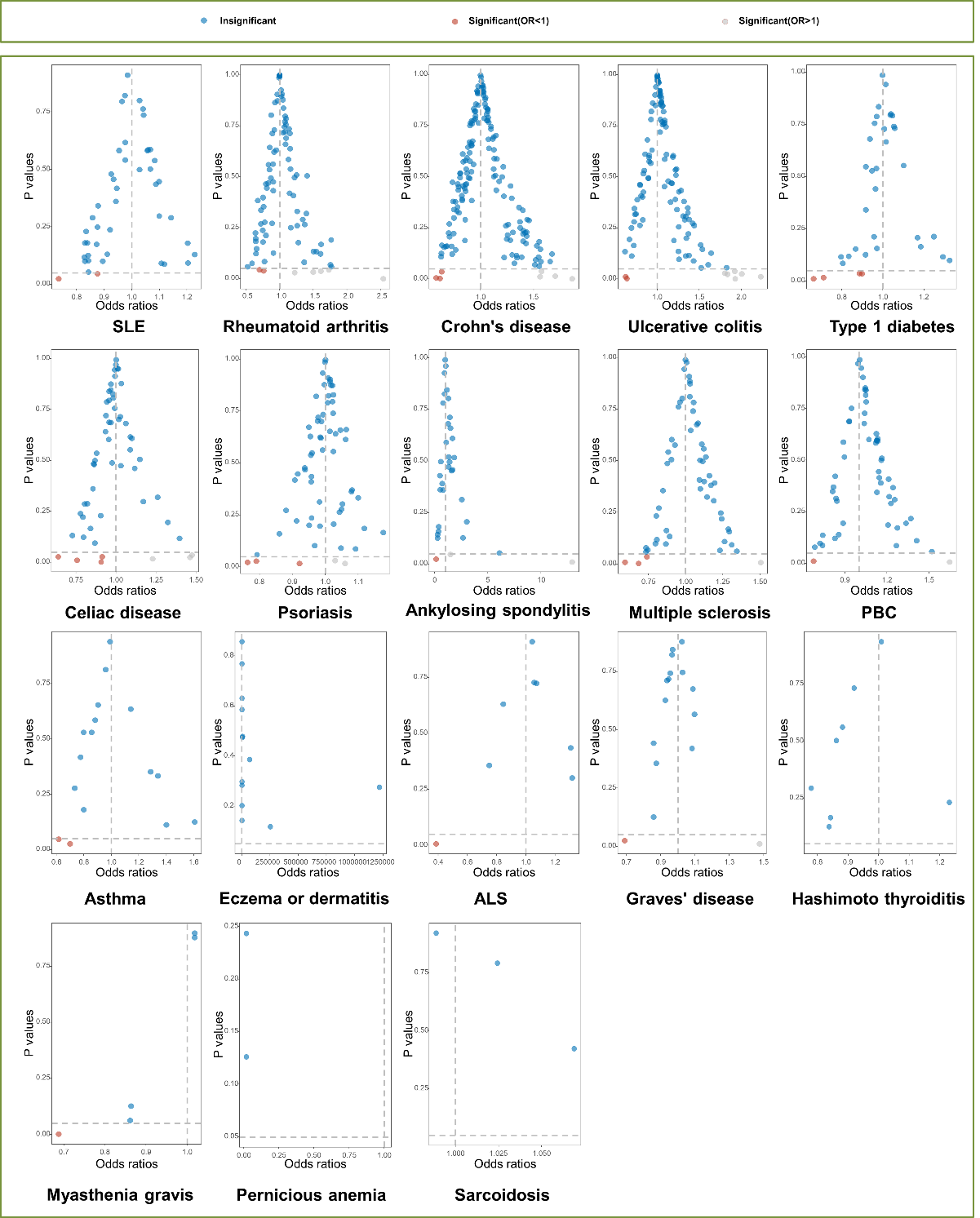


**Figure S15** The frost plots of the association between genetically predicted autoimmune diseases from FinnGen and glioma in the MR analysis. SLE, Systemic lupus erythematosus; MR, Mendelian randomization; PBC, Primary biliary cholangitis; ALS, Amyotrophic lateral sclerosis


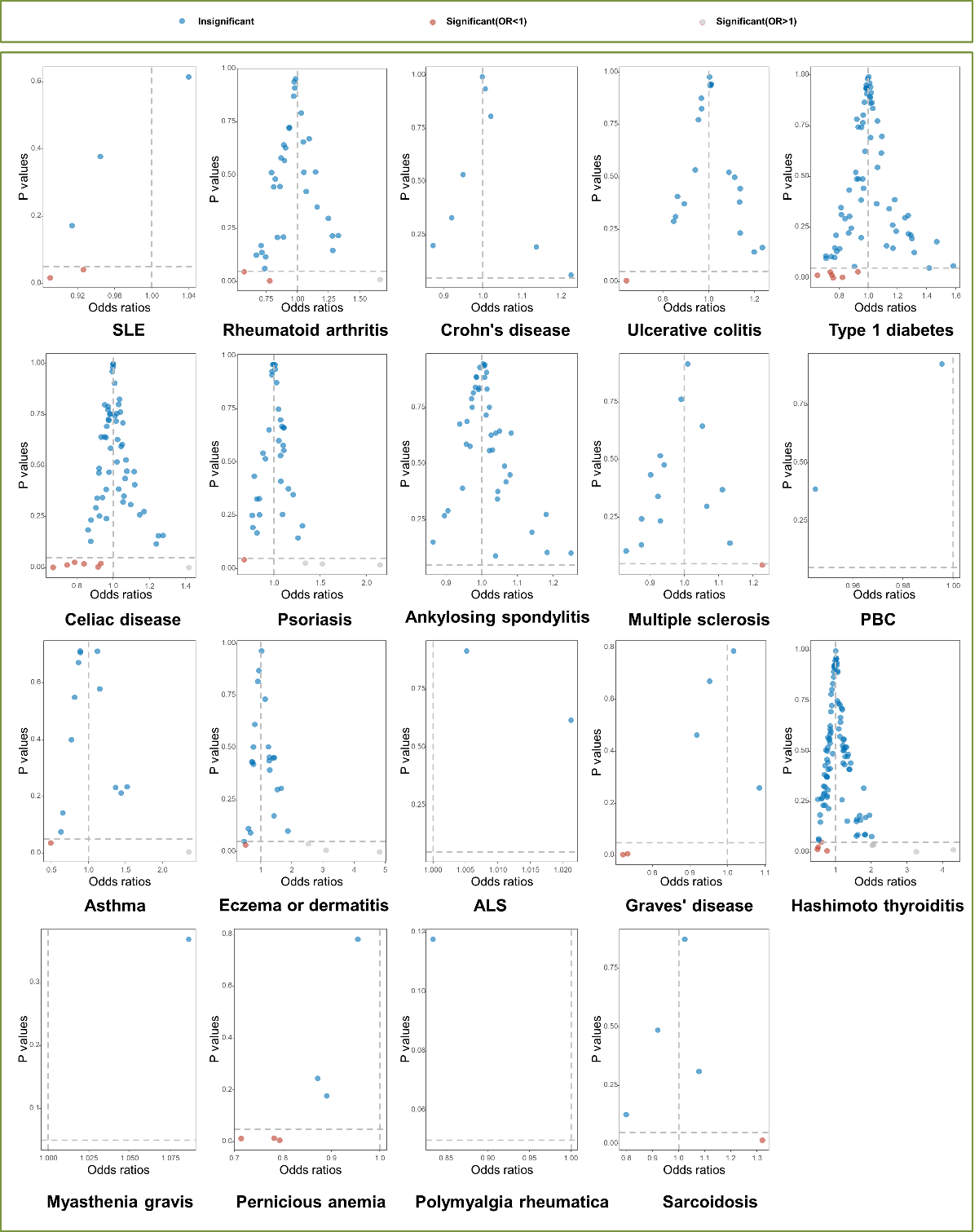


**Figure S19** The frost plots of the association between genetically predicted autoimmune diseases from FinnGen and LGG in the MR analysis. SLE, Systemic lupus erythematosus; MR, Mendelian randomization; PBC, Primary biliary cholangitis; ALS, Amyotrophic lateral sclerosis; LGG, lower-grade glioma


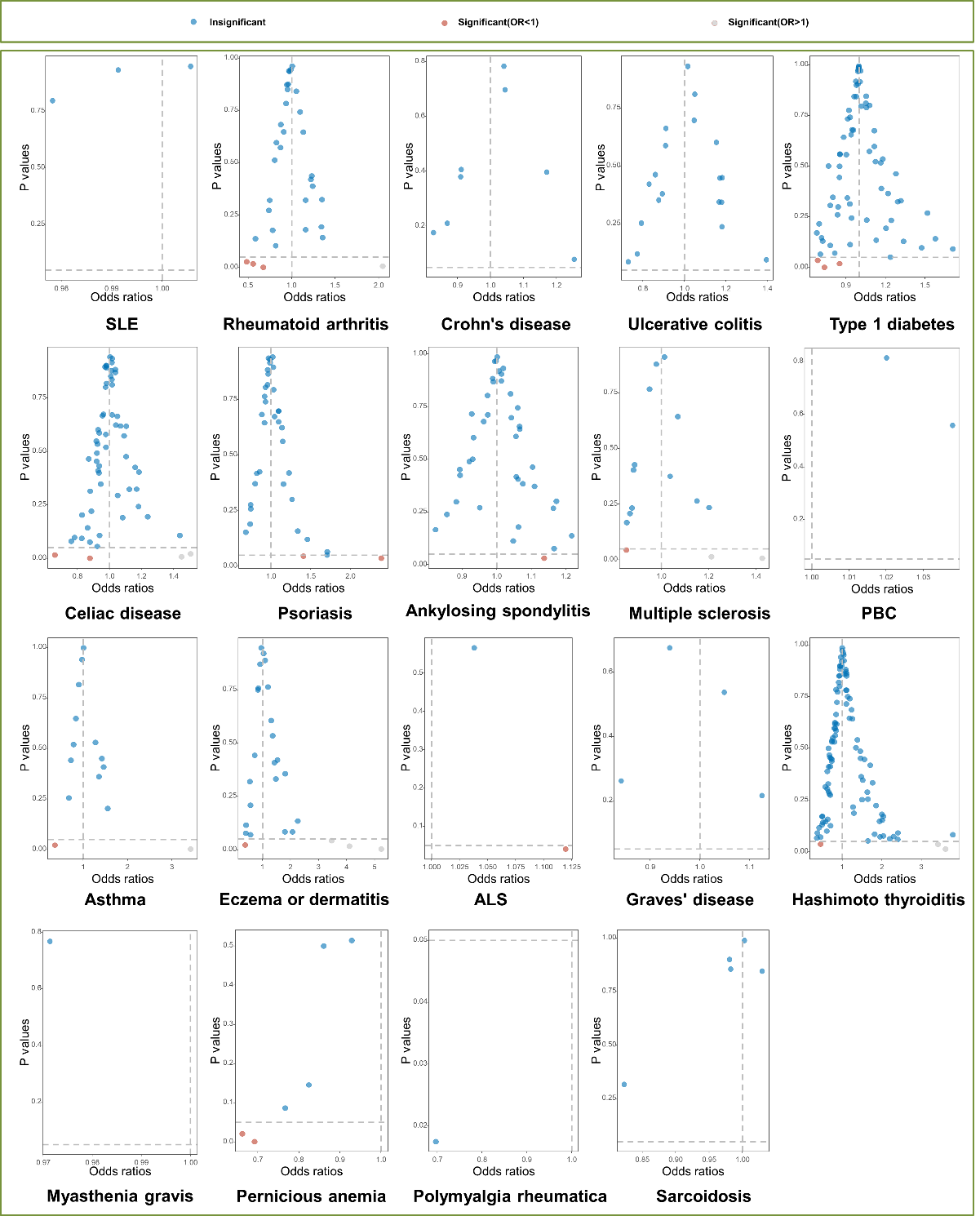


**Figure S23** The frost plots of the association between genetically predicted autoimmune diseases from FinnGen and GBM in the MR analysis. SLE, Systemic lupus erythematosus; MR, Mendelian randomization; GBM, glioblastoma; ALS, Amyotrophic lateral sclerosis


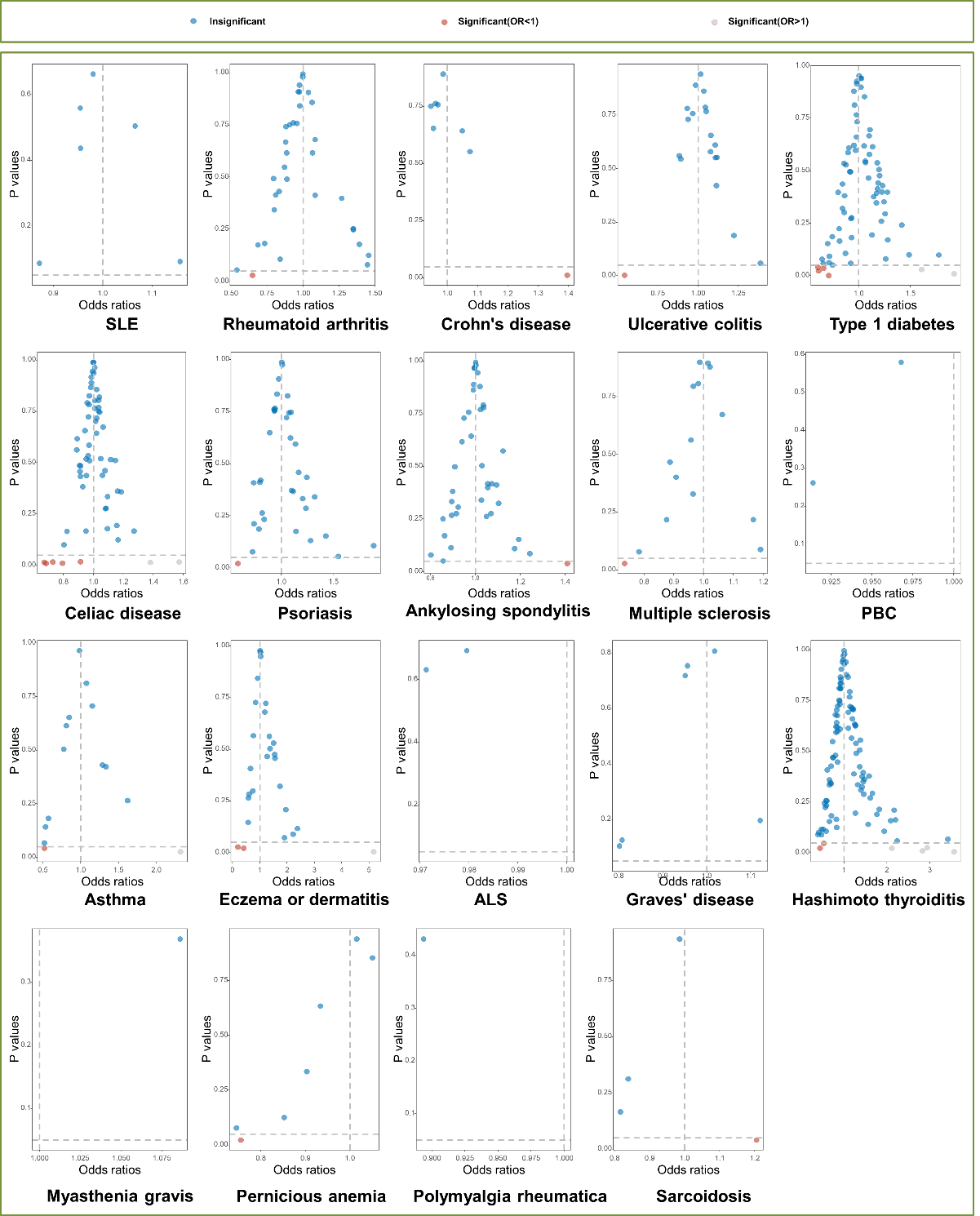

Supplement: Supplementary file 7 [file medi-104-e41815-s007.docx]
