# Supplementary material for: Comprehensive causal analysis between autoimmune diseases and glioma: A Mendelian randomization study
Source: Medicine (Baltimore). 2025 Mar 7;104(10):e41815. doi: 10.1097/MD.0000000000041815 (PMC11902947; doi:10.1097/MD.0000000000041815)

**Figure S25** The scatter plots of the association between genetically predicted glioma and autoimmune diseases from UKB in the reverse MR analysis. SLE, Systemic lupus erythematosus; MR, Mendelian randomization


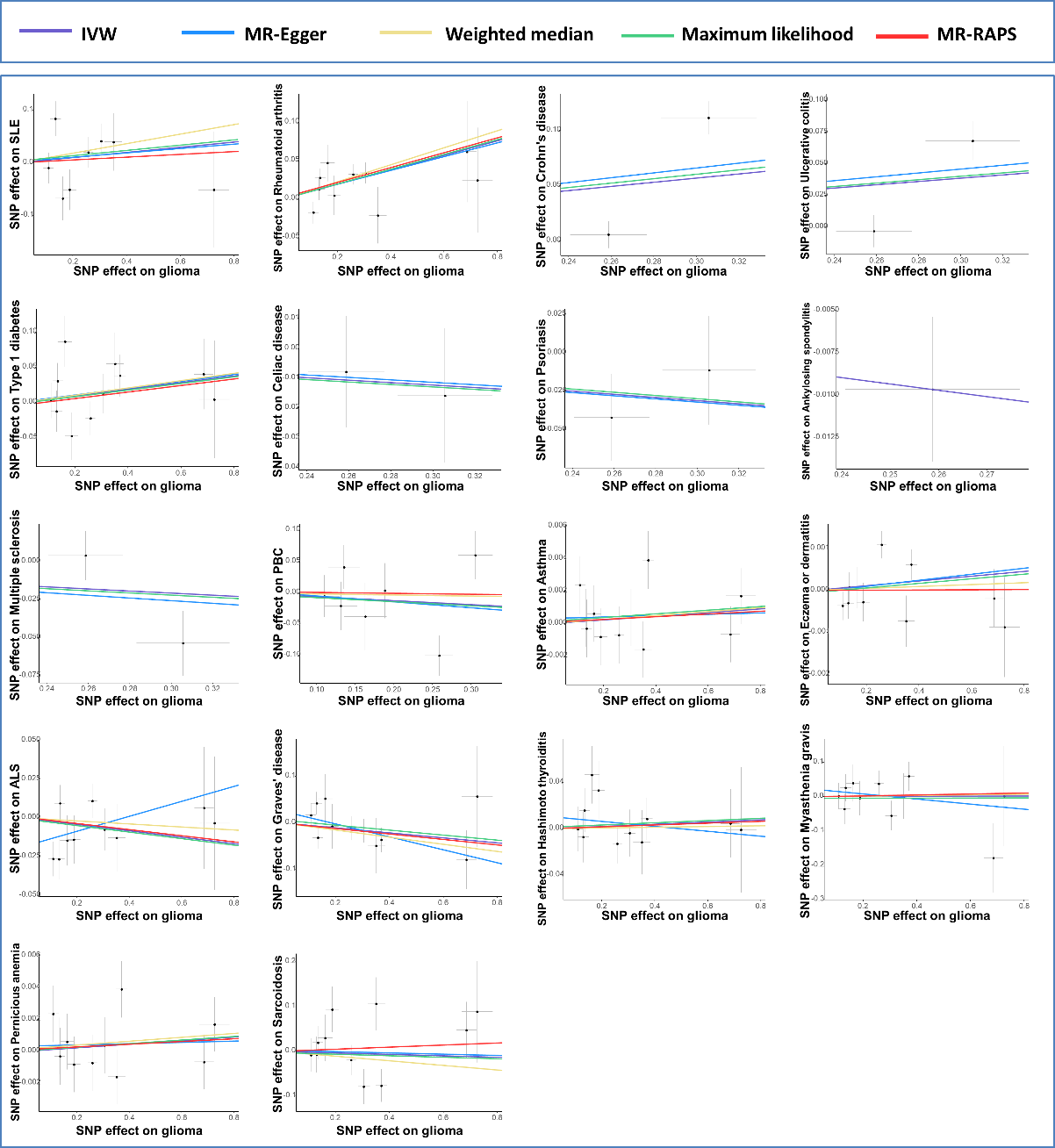


**Figure S29** The scatter plots of the association between genetically predicted LGG and autoimmune diseases from UKB in the reverse MR analysis. SLE, Systemic lupus erythematosus; MR, Mendelian randomization; LGG, lower-grade glioma


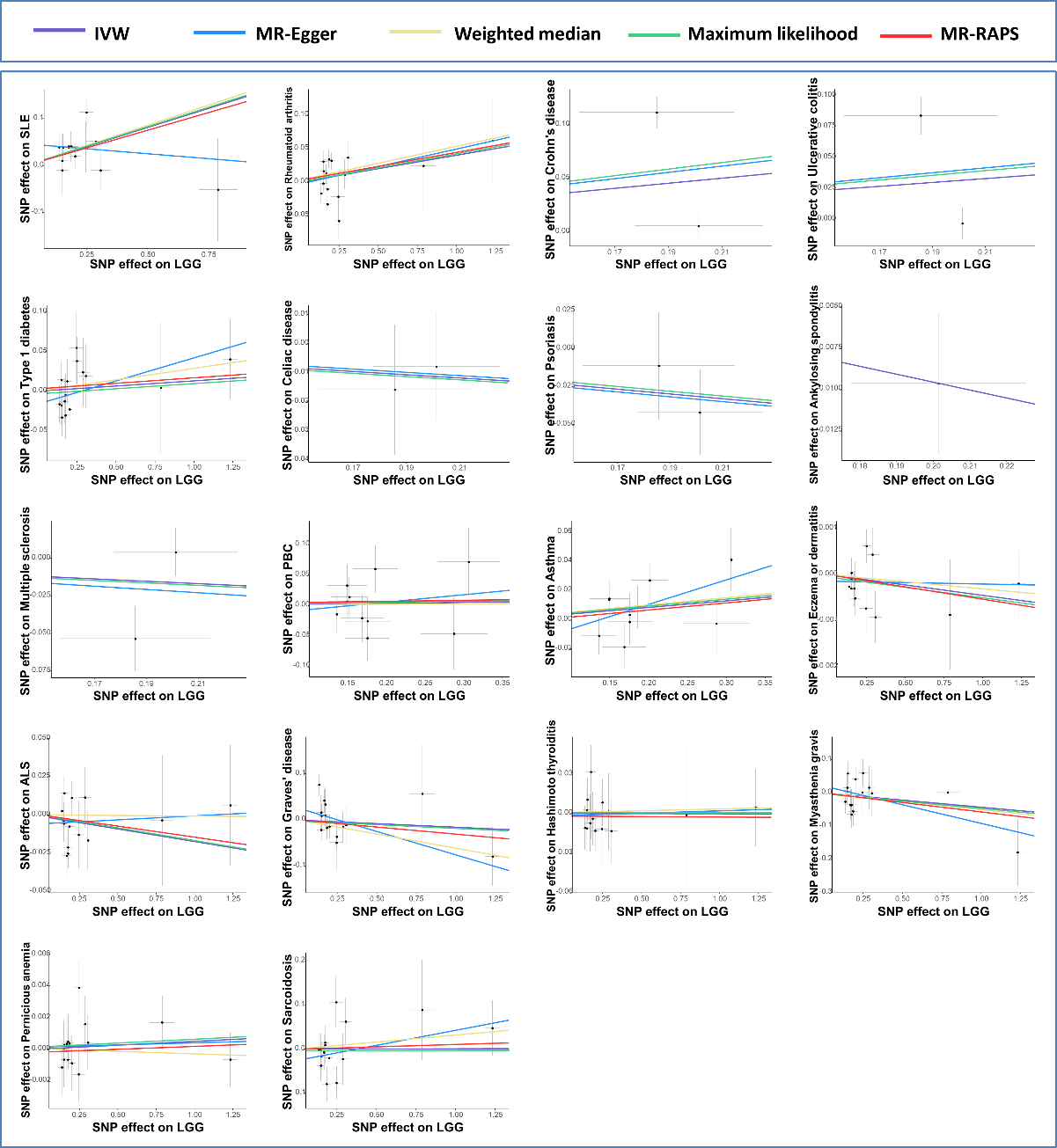


**Figure S33** The scatter plots of the association between genetically predicted GBM and autoimmune diseases from UKB in the reverse MR analysis. SLE, Systemic lupus erythematosus; MR, Mendelian randomization; GBM, glioblastoma


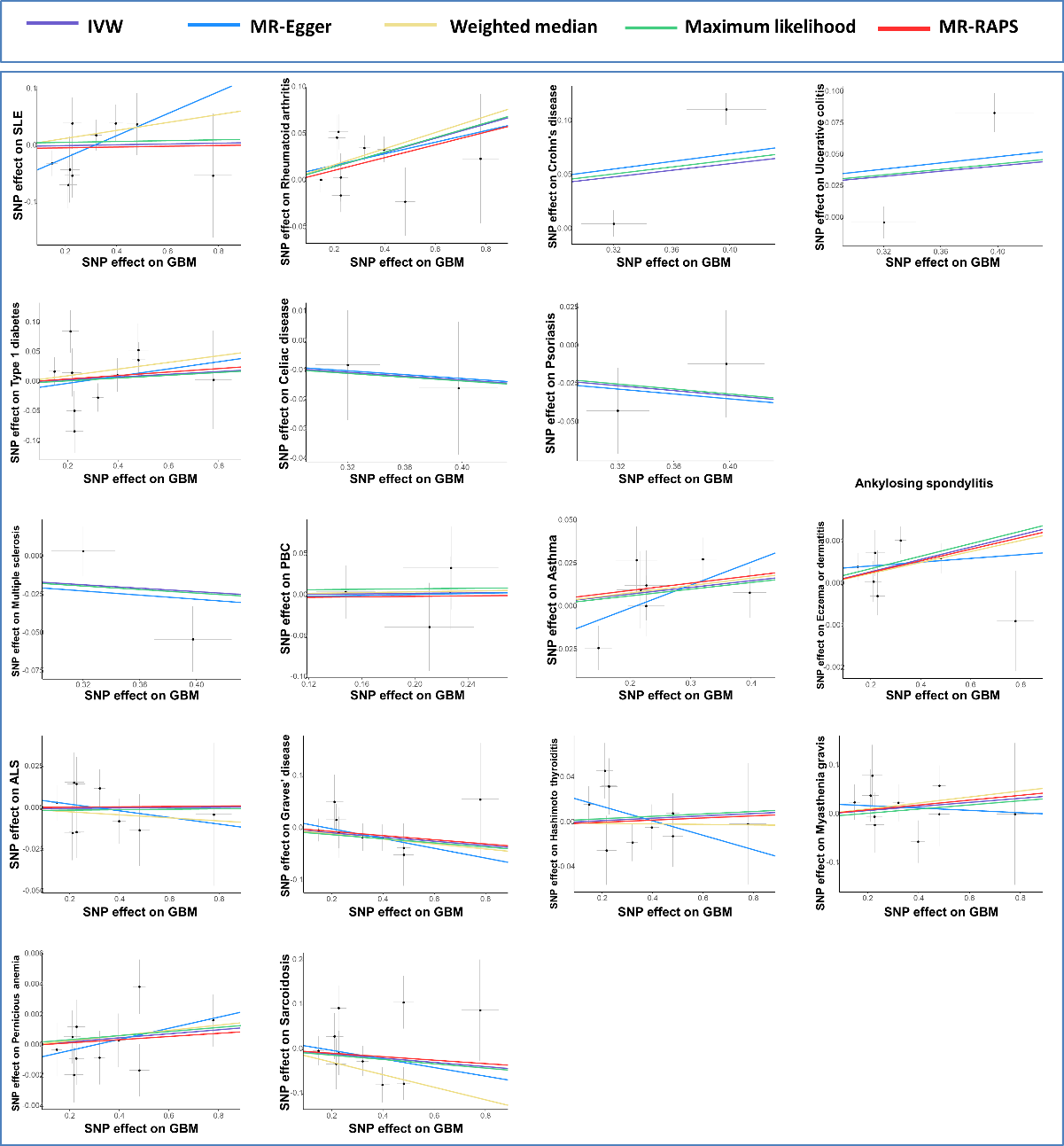


**Figure S37** The scatter plots of the association between genetically predicted glioma and autoimmune diseases from FinnGen in the reverse MR analysis. SLE, Systemic lupus erythematosus; MR, Mendelian randomization; ALS, Amyotrophic lateral sclerosis


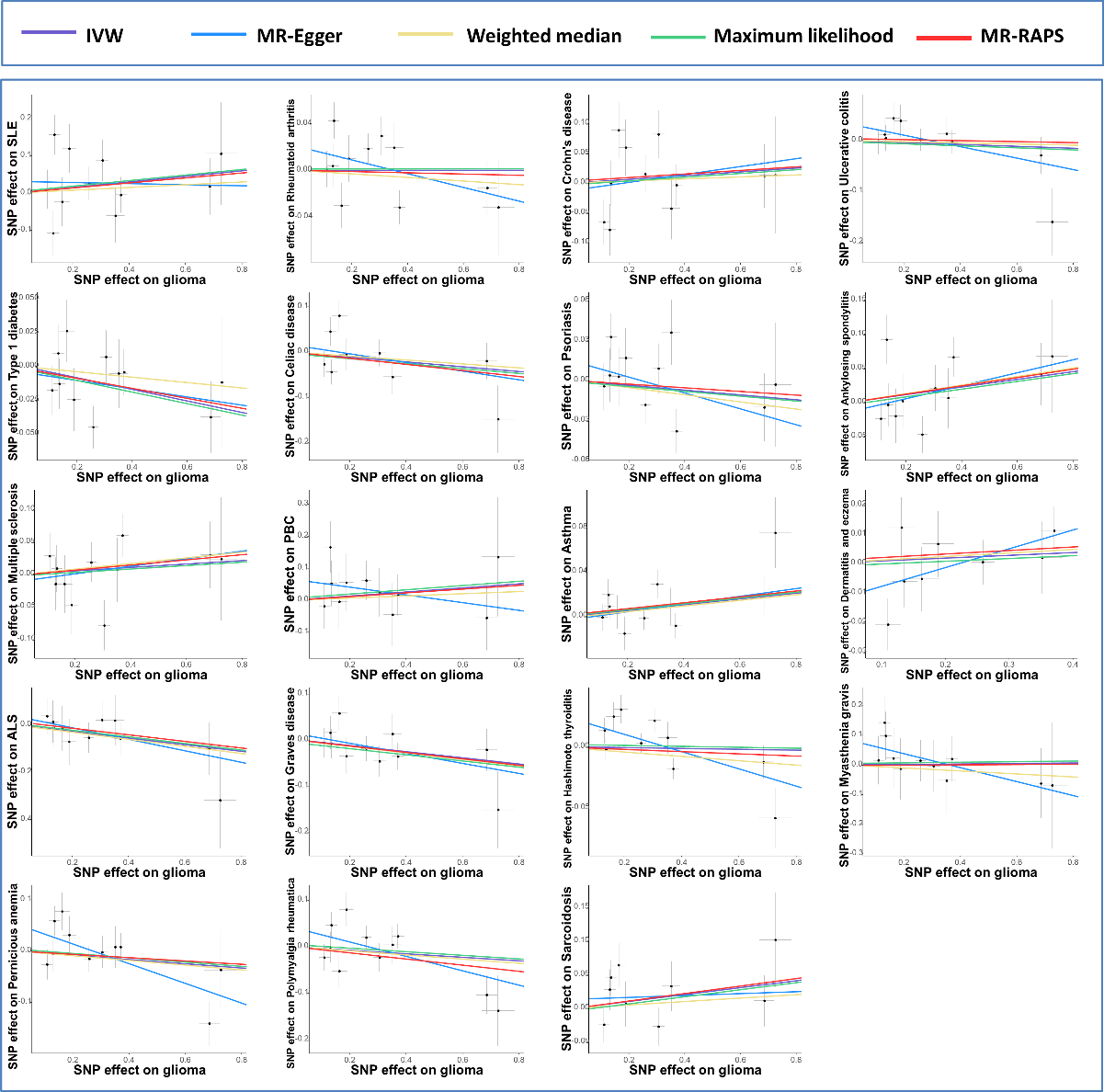


**Figure S41** The scatter plots of the association between genetically predicted LGG and autoimmune diseases from FinnGen in the reverse MR analysis. SLE, Systemic lupus erythematosus; MR, Mendelian randomization; ALS, Amyotrophic lateral sclerosis; LGG, lower-grade glioma


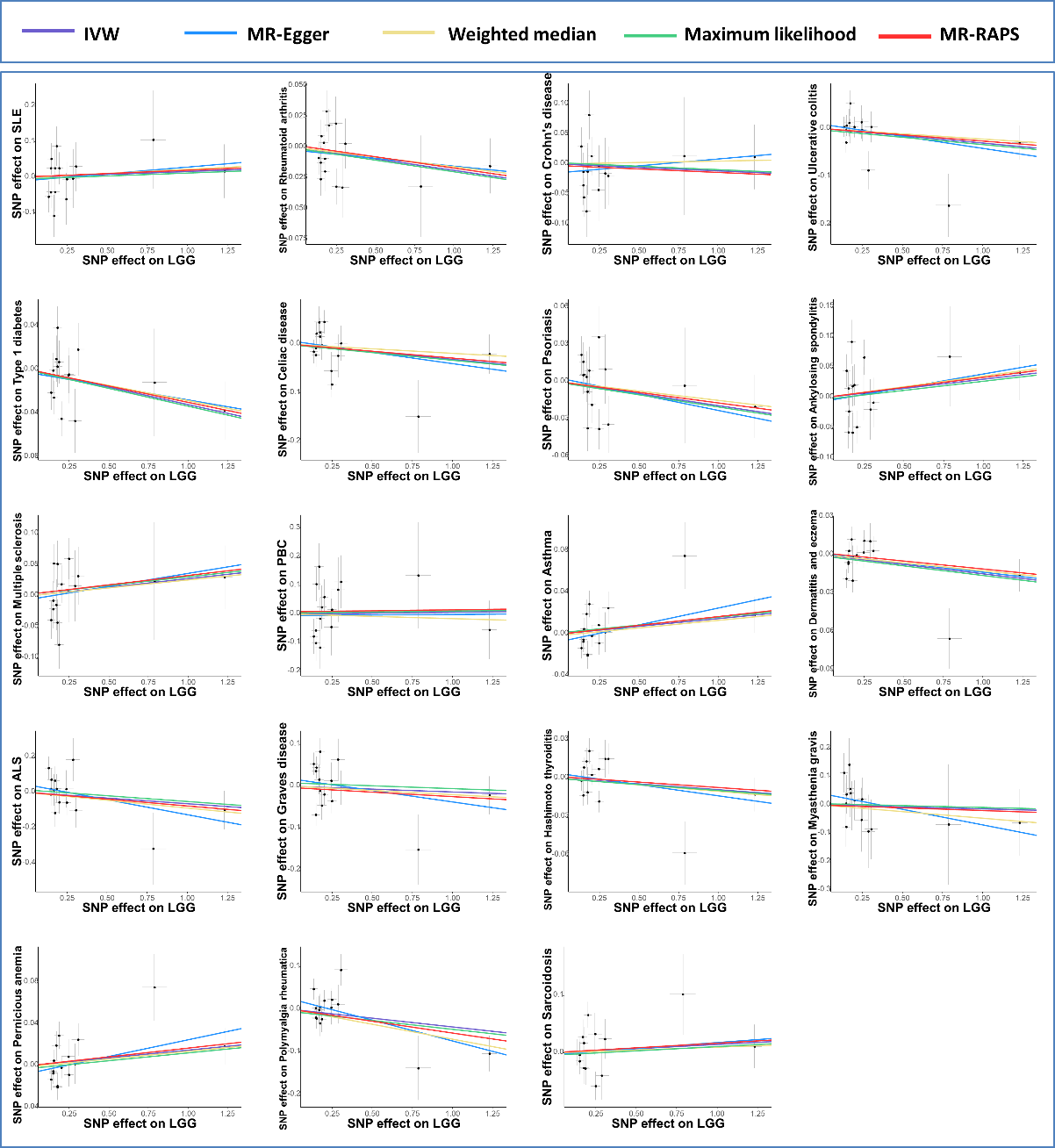


**Figure S45** The scatter plots of the association between genetically predicted GBM and autoimmune diseases from FinnGen in the reverse MR analysis. SLE, Systemic lupus erythematosus; MR, Mendelian randomization; ALS, Amyotrophic lateral sclerosis; GBM, glioblastoma


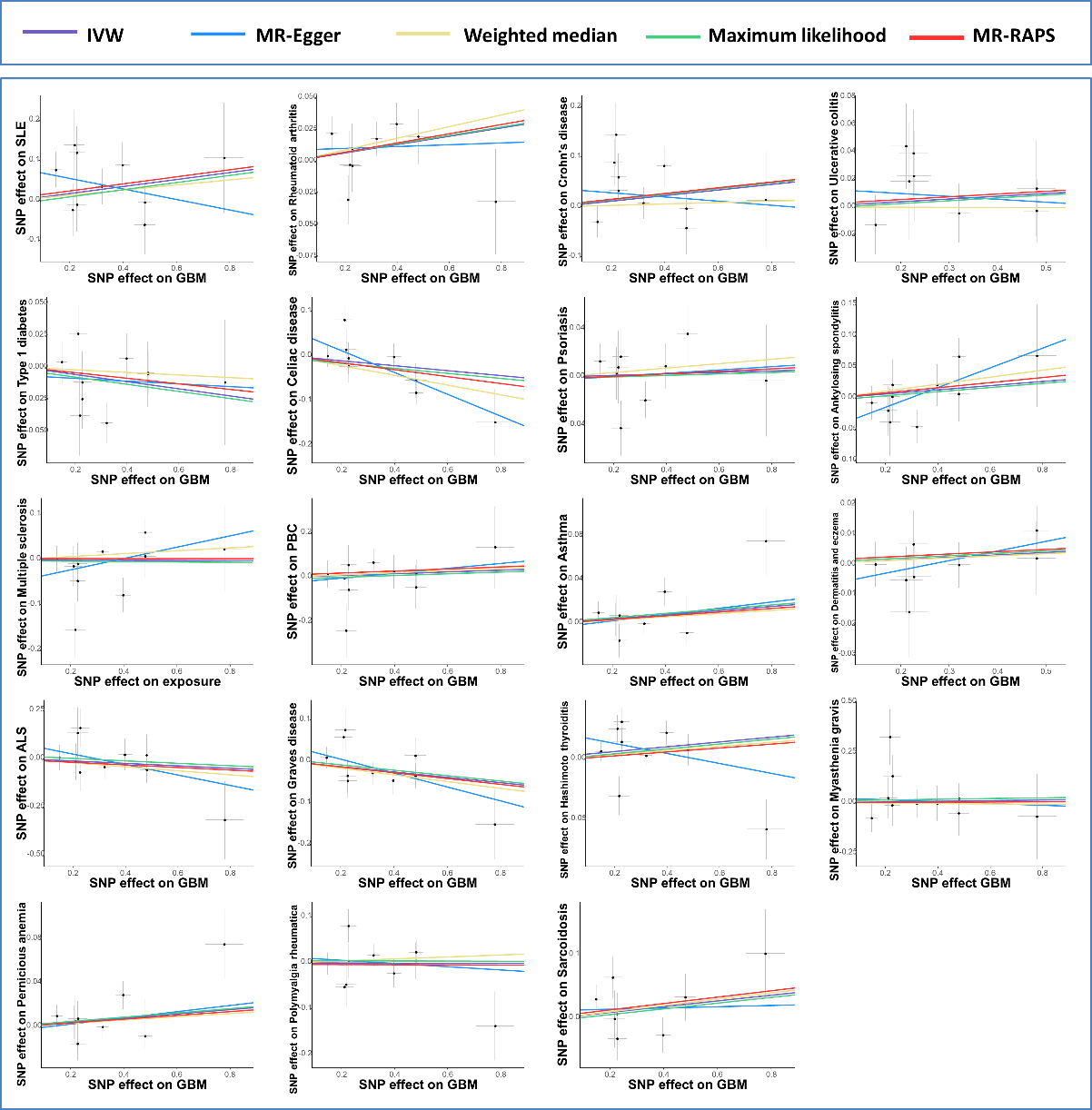

Supplement: Supplementary file 11 [file medi-104-e41815-s011.docx]
