# Supplementary material for: Comprehensive causal analysis between autoimmune diseases and glioma: A Mendelian randomization study
Source: Medicine (Baltimore). 2025 Mar 7;104(10):e41815. doi: 10.1097/MD.0000000000041815 (PMC11902947; doi:10.1097/MD.0000000000041815)

**Figure S26** The funnel plots of the association between genetically predicted glioma and autoimmune diseases from UKB in the reverse MR analysis. SLE, Systemic lupus erythematosus; MR, Mendelian randomization; PBC, Primary biliary cholangitis; ALS, Amyotrophic lateral sclerosis


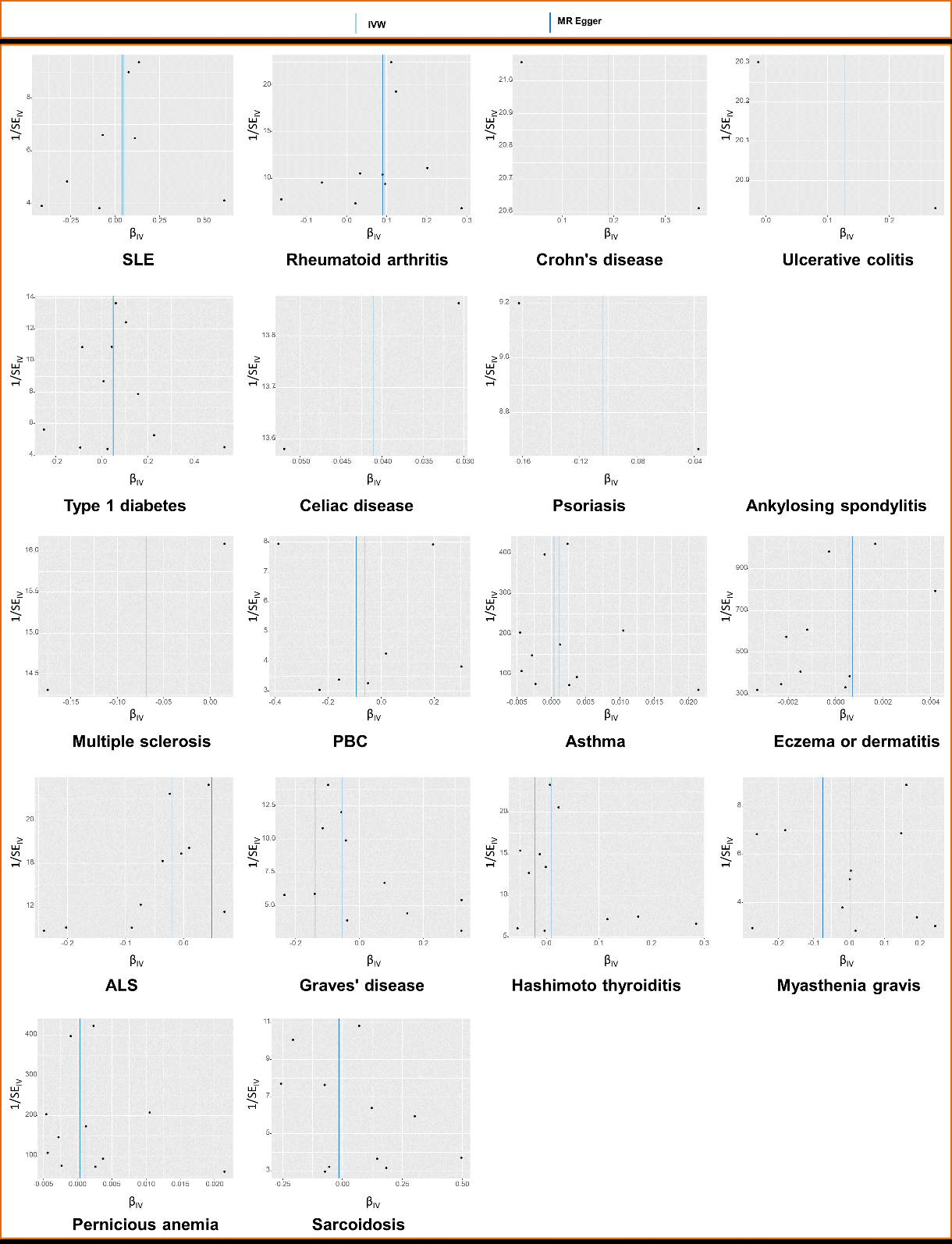


**Figure S30** The funnel plots of the association between genetically predicted LGG and autoimmune diseases from UKB in the reverse MR analysis. SLE, Systemic lupus erythematosus; MR, Mendelian randomization; PBC, Primary biliary cholangitis; ALS, Amyotrophic lateral sclerosis; LGG, lower-grade glioma


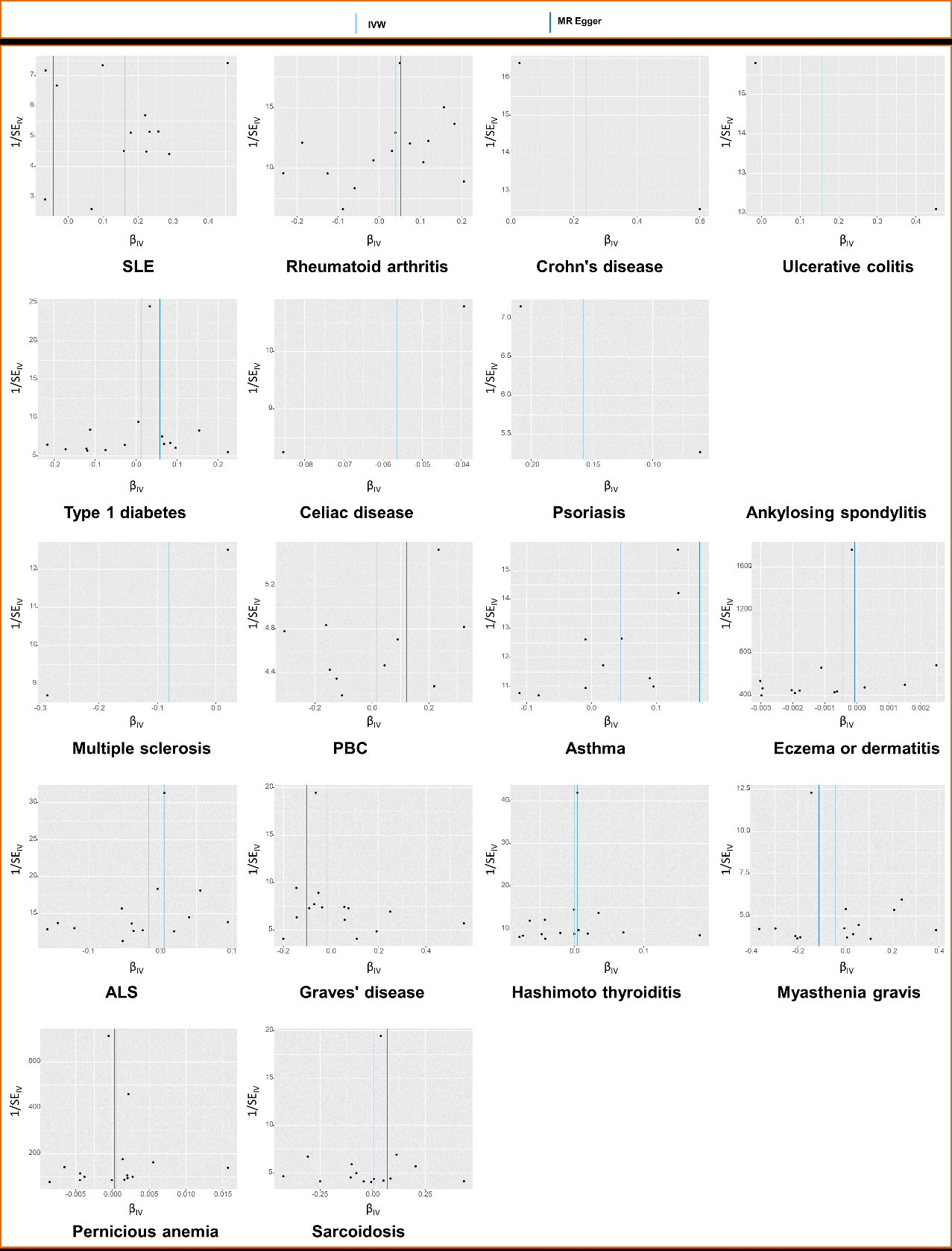


**Figure S34** The funnel plots of the association between genetically predicted GBM and autoimmune diseases from UKB in the reverse MR analysis. SLE, Systemic lupus erythematosus; MR, Mendelian randomization; PBC, Primary biliary cholangitis; ALS, Amyotrophic lateral sclerosis; GBM, glioblastoma


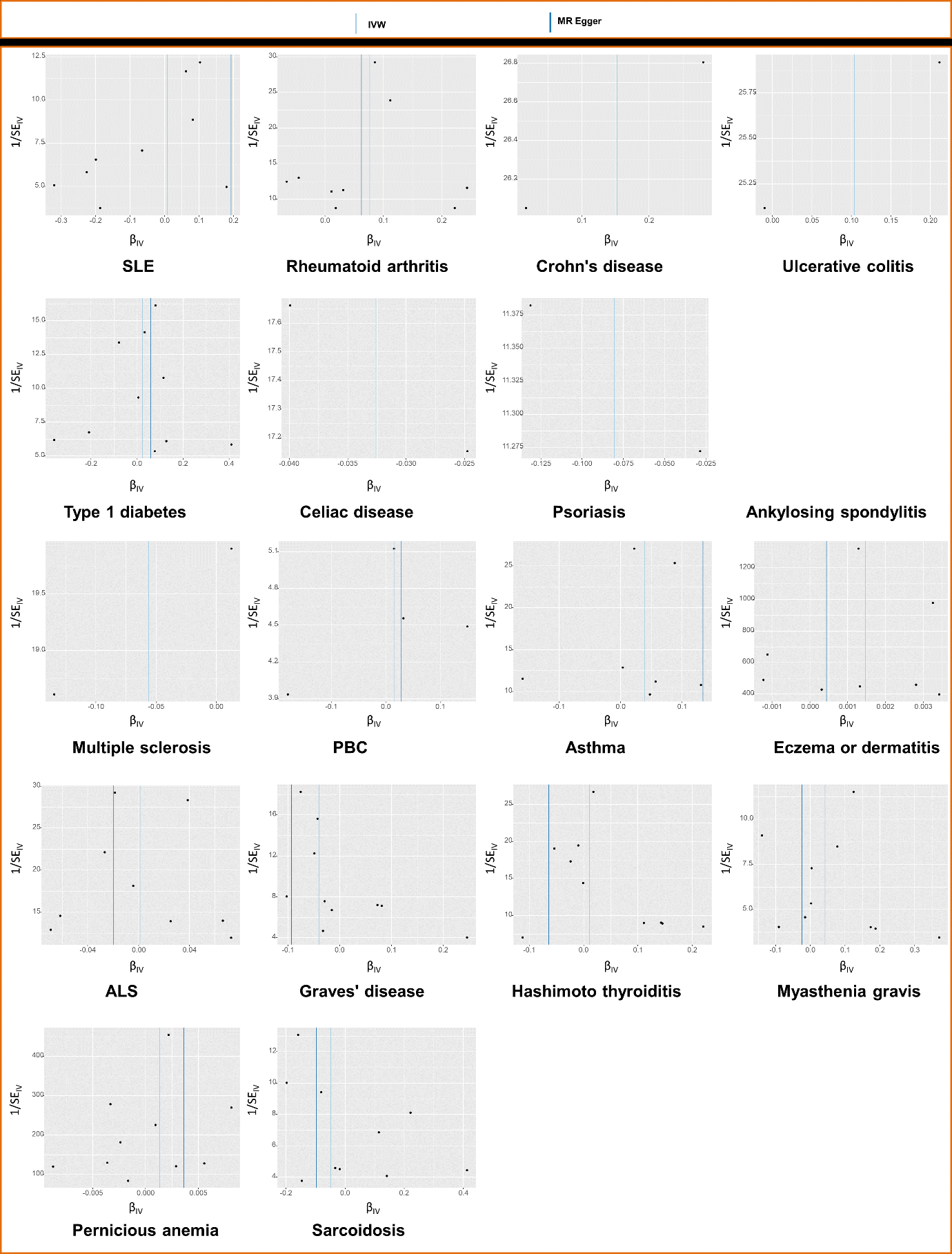


**Figure S38** The funnel plots of the association between genetically predicted glioma and autoimmune diseases from FinnGen in the reverse MR analysis. SLE, Systemic lupus erythematosus; MR, Mendelian randomization; PBC, Primary biliary cholangitis; ALS, Amyotrophic lateral sclerosis


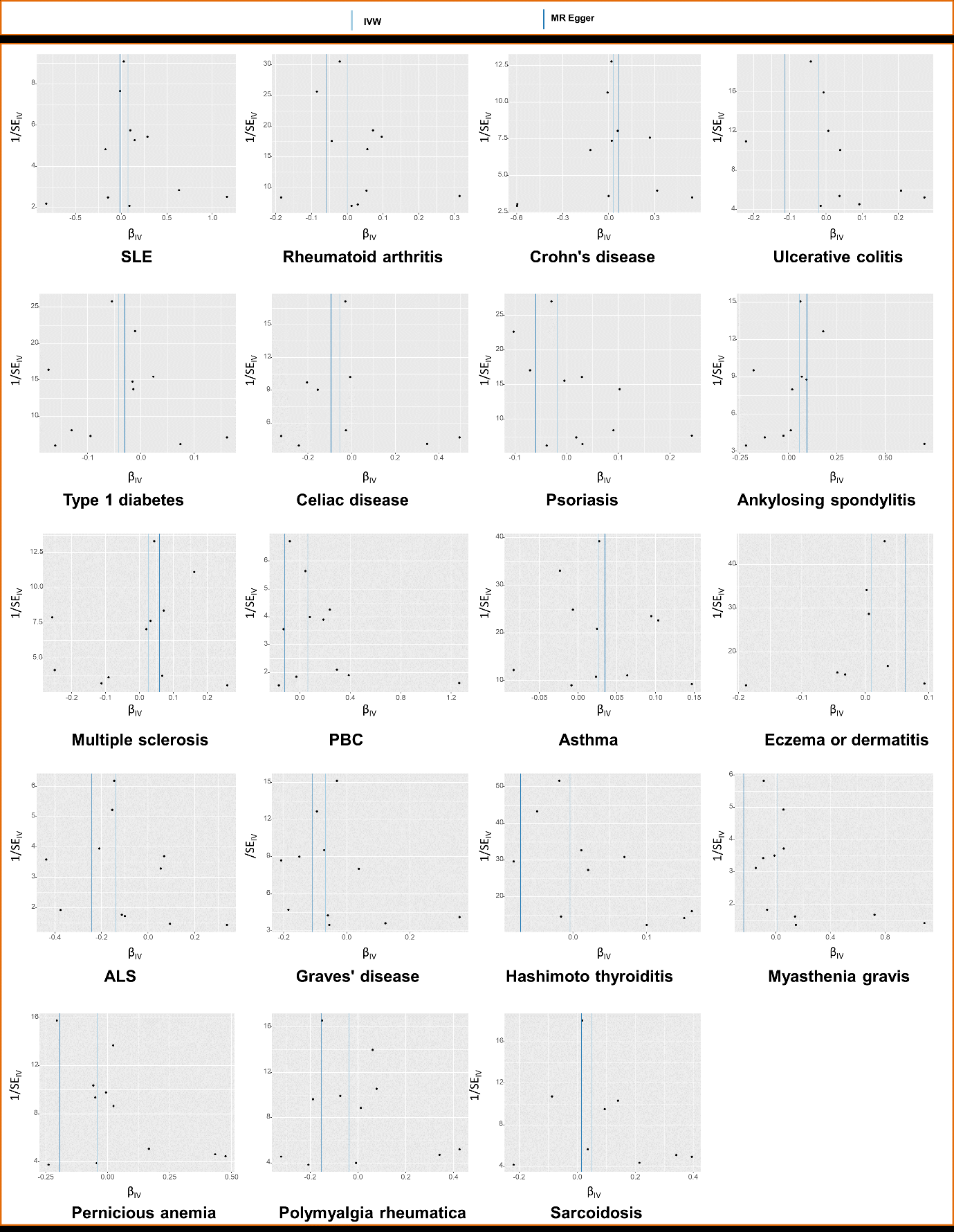


**Figure S42** The funnel plots of the association between genetically predicted LGG and autoimmune diseases from FinnGen in the reverse MR analysis. SLE, Systemic lupus erythematosus; MR, Mendelian randomization; PBC, Primary biliary cholangitis; ALS, Amyotrophic lateral sclerosis; LGG, lower-grade glioma


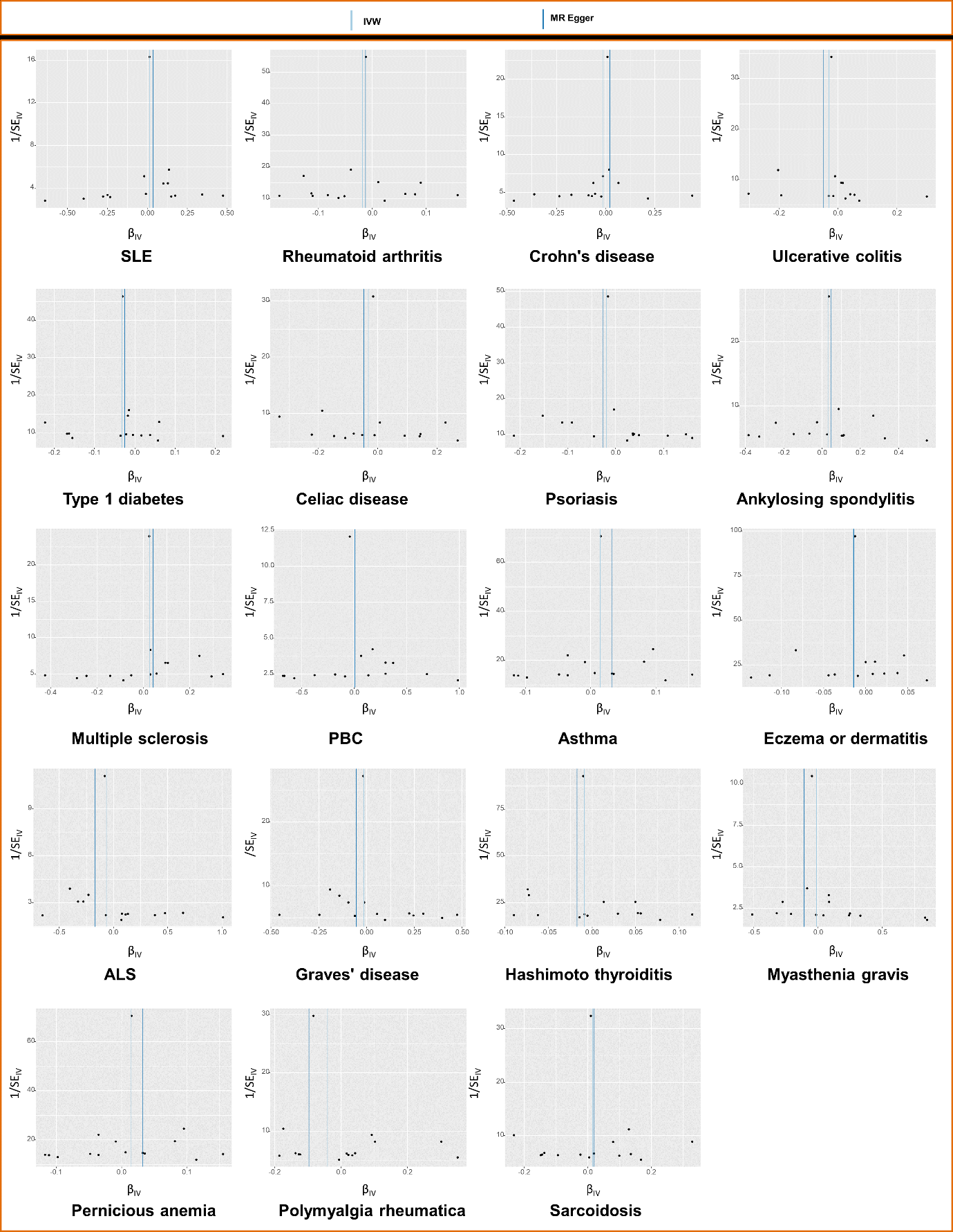


**Figure S46** The funnel plots of the association between genetically predicted GBM and autoimmune diseases from FinnGen in the reverse MR analysis. SLE, Systemic lupus erythematosus; MR, Mendelian randomization; PBC, Primary biliary cholangitis; ALS, Amyotrophic lateral sclerosis; GBM, glioblastoma


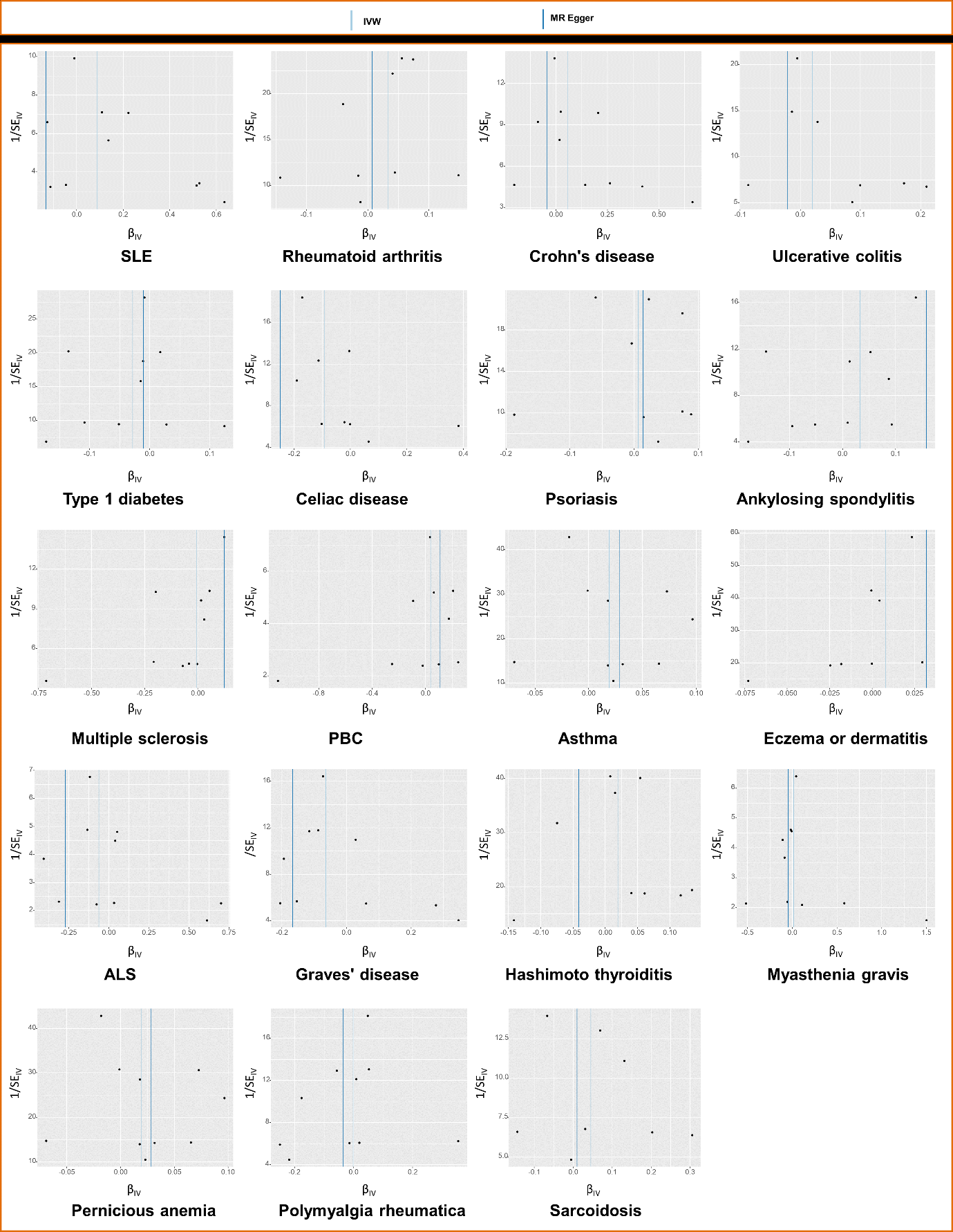

Supplement: Supplementary file 12 [file medi-104-e41815-s012.docx]
