# Supplementary material for: Comprehensive causal analysis between autoimmune diseases and glioma: A Mendelian randomization study
Source: Medicine (Baltimore). 2025 Mar 7;104(10):e41815. doi: 10.1097/MD.0000000000041815 (PMC11902947; doi:10.1097/MD.0000000000041815)
Supplement: Supplementary file 13 [file medi-104-e41815-s013.docx]

**Figure S27** The frost plots of the association between genetically predicted glioma and autoimmune diseases from UKB in the reverse MR analysis. SLE, Systemic lupus erythematosus; MR, Mendelian randomization; PBC, Primary biliary cholangitis; ALS, Amyotrophic lateral sclerosis


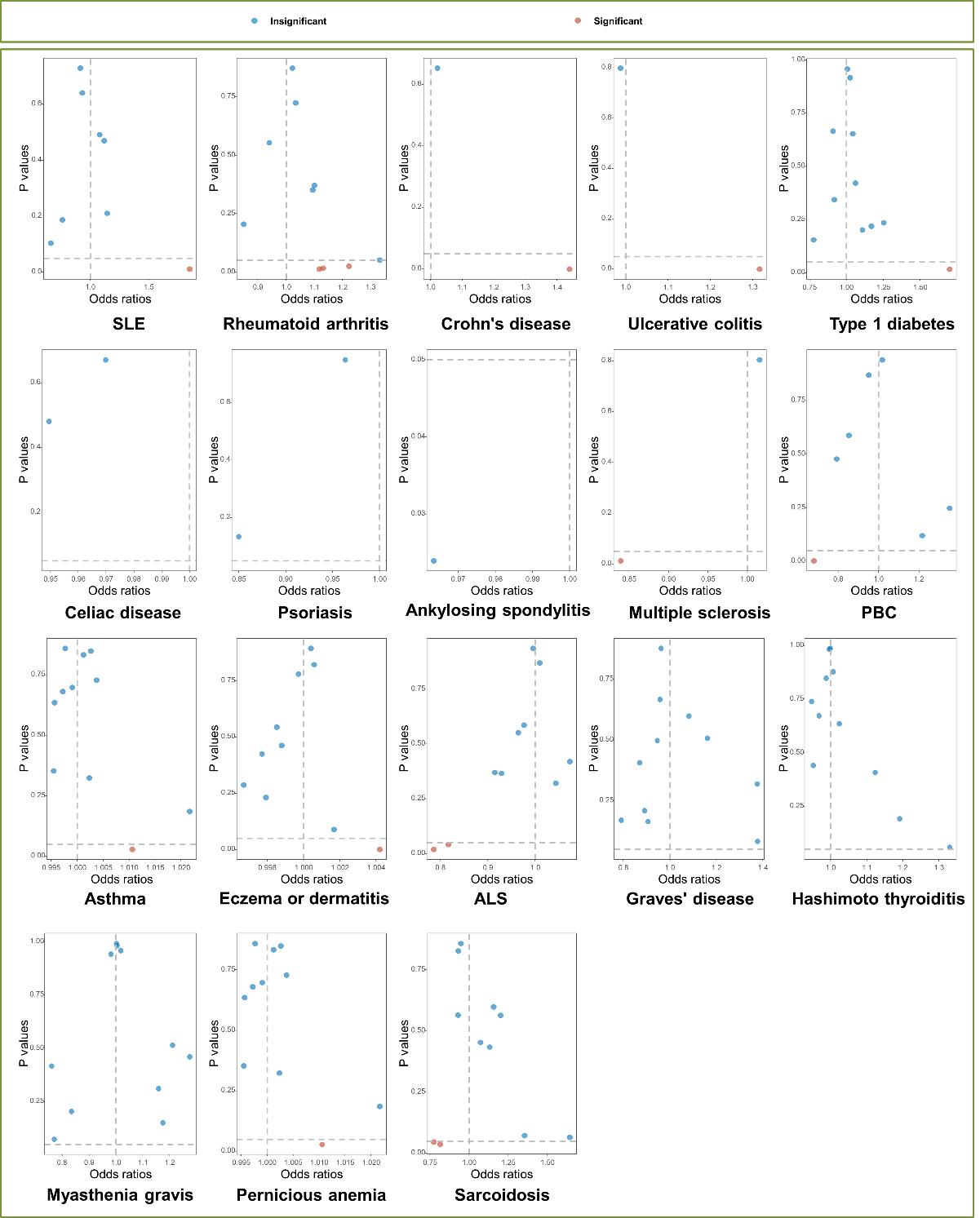


**Figure S31** The frost plots of the association between genetically predicted LGG and autoimmune diseases from UKB in the reverse MR analysis. SLE, Systemic lupus erythematosus; MR, Mendelian randomization; PBC, Primary biliary cholangitis; ALS, Amyotrophic lateral sclerosis; LGG, lower-grade glioma


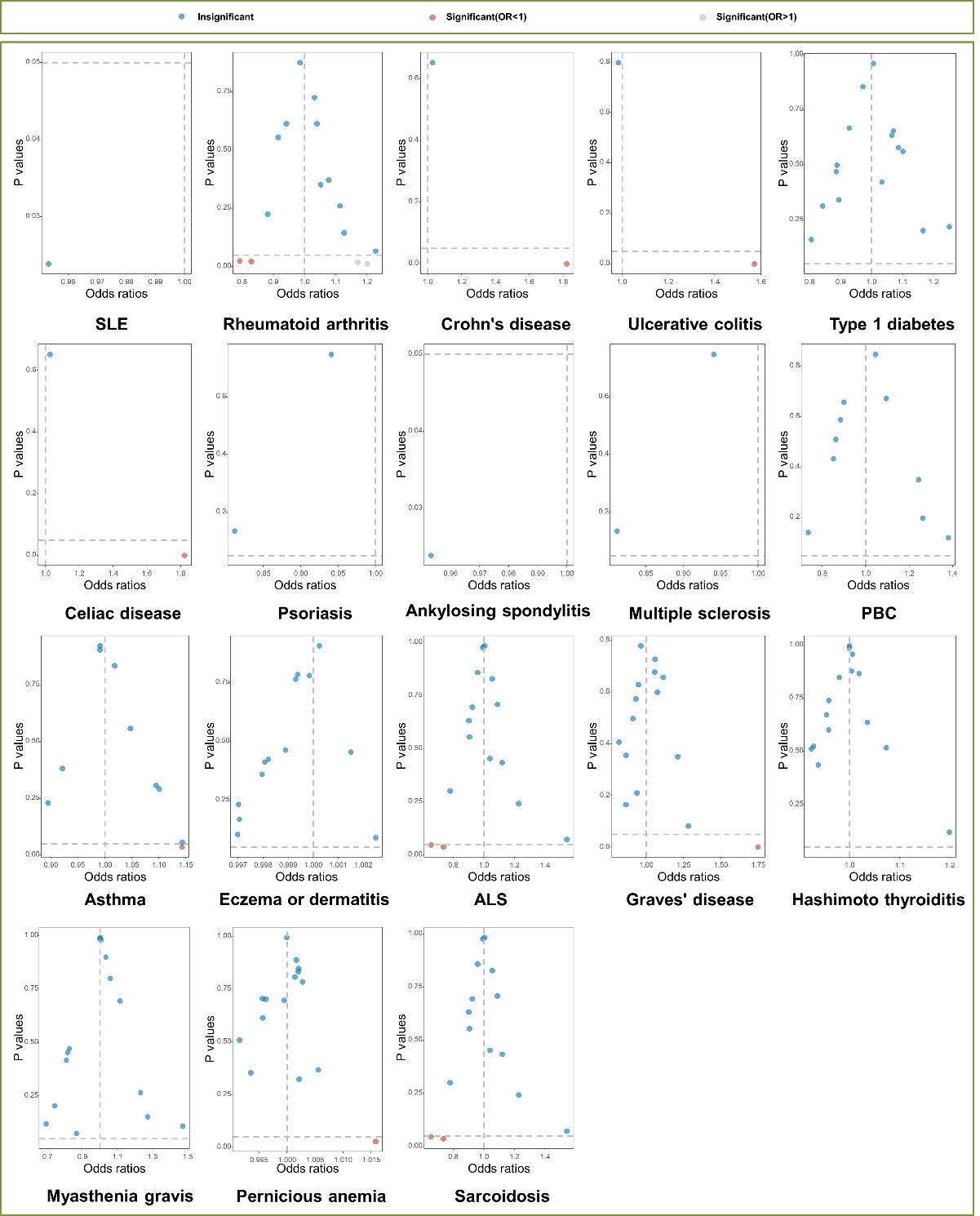


**Figure S35** The frost plots of the association between genetically predicted GBM and autoimmune diseases from UKB in the reverse MR analysis. SLE, Systemic lupus erythematosus; MR, Mendelian randomization; PBC, Primary biliary cholangitis; ALS, Amyotrophic lateral sclerosis; GBM, glioblastoma


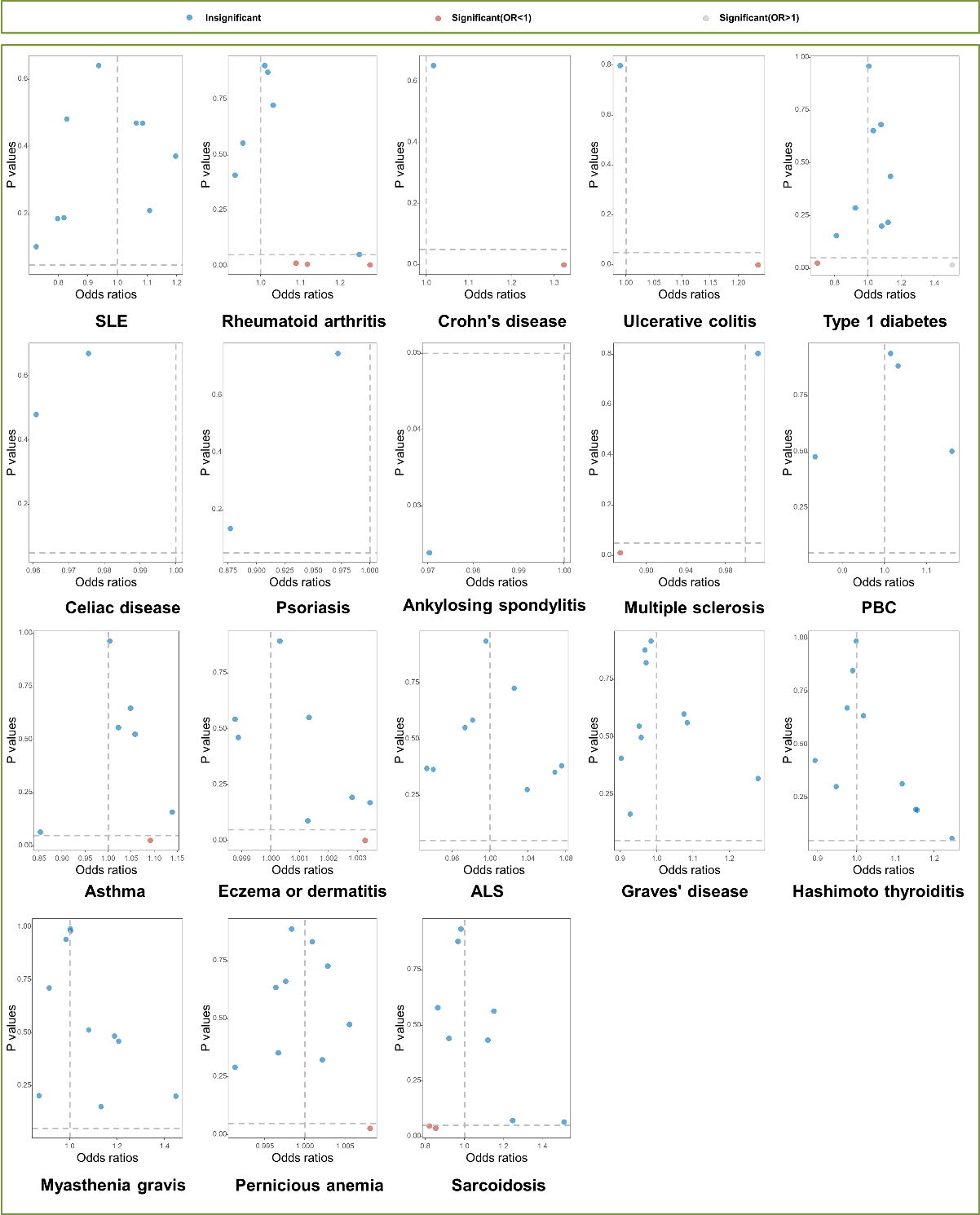


**Figure S39** The frost plots of the association between genetically predicted glioma and autoimmune diseases from FinnGen in the reverse MR analysis. SLE, Systemic lupus erythematosus; MR, Mendelian randomization; ALS, Amyotrophic lateral sclerosis


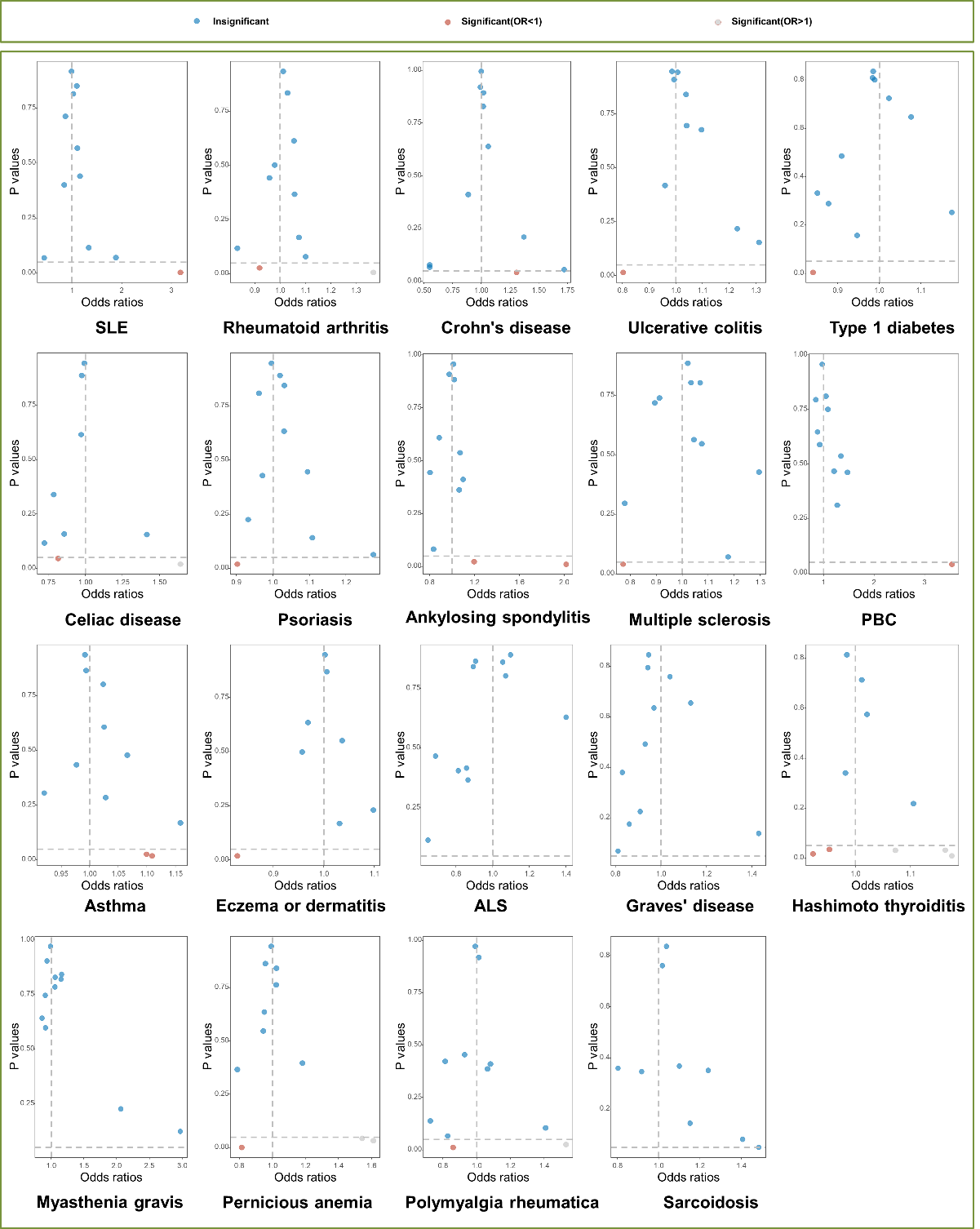


**Figure S43** The frost plots of the association between genetically predicted LGG and autoimmune diseases from FinnGen in the reverse MR analysis. SLE, Systemic lupus erythematosus; MR, Mendelian randomization; ALS, Amyotrophic lateral sclerosis; LGG, lower-grade glioma


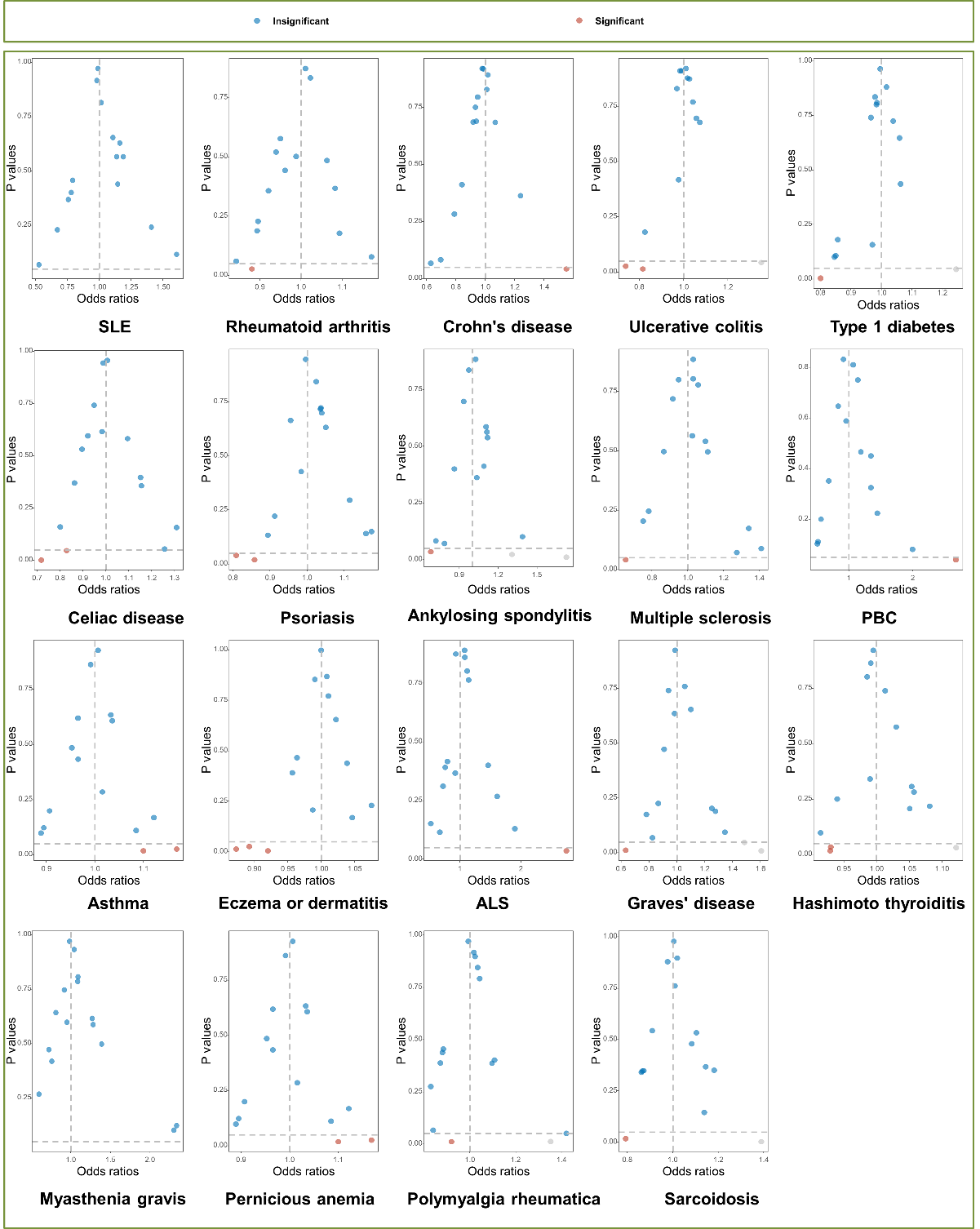


**Figure S47** The frost plots of the association between genetically predicted GBM and autoimmune diseases from FinnGen in the reverse MR analysis. SLE, Systemic lupus erythematosus; MR, Mendelian randomization; ALS, Amyotrophic lateral sclerosis; GBM, glioblastoma


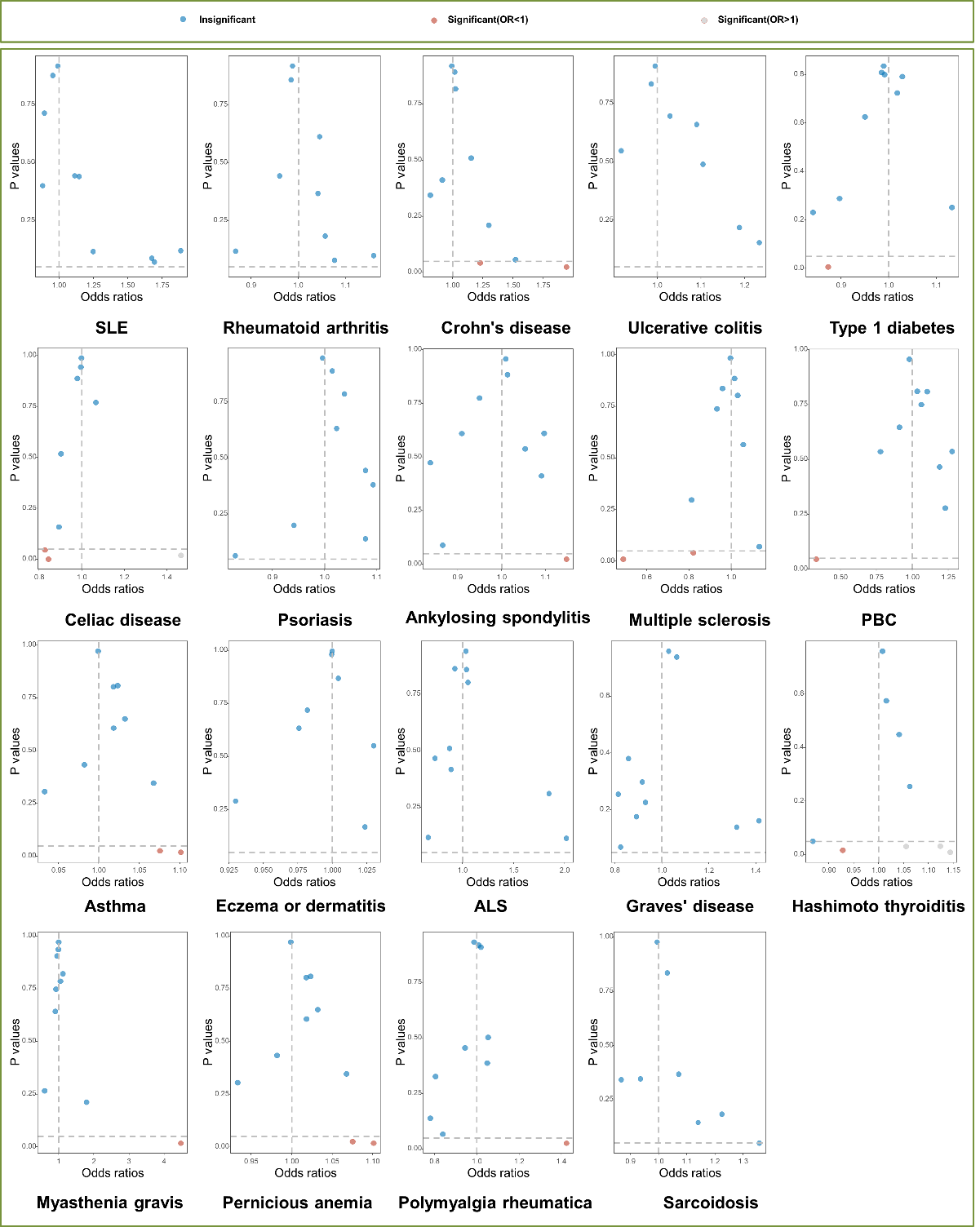


V
